# Classical analogue to driven quantum bits based on macroscopic pendula

**DOI:** 10.1038/s41598-023-45118-y

**Published:** 2023-10-26

**Authors:** Heribert Lorenz, Sigmund Kohler, Anton Parafilo, Mikhail Kiselev, Stefan Ludwig

**Affiliations:** 1 Fakultät für Physik, Center for NanoScience (CeNS), Ludwig-Maximilians-Universität (LMU), 80539 München, Germany; 2 Instituto de Ciencia de Materiales de Madrid, CSIC, 28049 Madrid, Spain; 3 Center for Theoretical Physics of Complex Systems (PCS), Institute for Basic Science (IBS), Expo-ro 55, Yuseong-gu, Daejeon, 34126 Korea; 4 The Abdus Salam International Centre for Theoretical Physics (ICTP), Strada Costiera 11, 34151 Trieste, Italy; 5 Paul-Drude-Institut für Festkörperelektronik (PDI), Leibniz-Institut im Forschungsverbund Berlin e.V., Hausvogteiplatz 5-7, 10117 Berlin, Germany

**Keywords:** Physics, Quantum physics

## Abstract

Quantum mechanics increasingly penetrates modern technologies but, due to its non-deterministic nature seemingly contradicting our classical everyday world, our comprehension often stays elusive. Arguing along the correspondence principle, classical mechanics is often seen as a theory for large systems where quantum coherence is completely averaged out. Surprisingly, it is still possible to reconstruct the coherent dynamics of a quantum bit (qubit) by using a classical model system. This classical-to-quantum analogue is based on wave mechanics, which applies to both, the classical and the quantum world. In this spirit we investigate the dynamics of macroscopic physical pendula with a modulated coupling. As a proof of principle, we demonstrate full control of our one-to-one analogue to a qubit by realizing Rabi oscillations, Landau-Zener transitions and Landau-Zener-Stückelberg-Majorana interferometry. Our classical qubit demonstrator can help comprehending and developing useful quantum technologies.

Quantum technology already has a drastic impact on society. This development presently accelerates with our growing ability to harvest coherent quantum dynamics for engineering game changing devices such as quantum computers or a quantum internet. At the same time, while the mathematical framework of quantum mechanics can be considered complete, fundamental aspects of the underlying physics, even on the level of only few qubits are outside our empirical world. In this situation, classical model systems capable of enlightening the often elusive coherent dynamics of quantum systems may prove very useful^1^. This approach might be fundamentally questioned due to a central paradigm of quantum dynamics, which is its probabilistic nature in contrast to the deterministic classical equation of motion (EOM). Nevertheless, besides non-determinism and non-locality, wave properties and the superposition principle being central elements of quantum mechanics appear also in classical physics. For example, the quantum mechanical double slit experiment may be visualized with classical water waves. Such similarities and the non-deterministic and non-local nature of quantum mechanics lead to the unsuccessful efforts of replacing quantum mechanics by hidden variable theories. These, in turn, motivated John Bell’s works really paving the way for quantum technology applications by putting the dynamics and measurement of quantum mechanical systems in a clear perspective to a classical description^2,3^. While the non-deterministic nature of quantum mechanics does not concern the coherent dynamics of a quantum system, it dominates the quantum measurement which cannot have a classical analogue^3^. Here we visualize the dynamics of one of the most basic quantum systems, a qubit, by physical macroscopic pendula.

Classical dynamics can generally be formulated in terms of second-order, non-linear and inhomogeneous differential equations, while non relativistic quantum mechanics is based on the first order, homogeneous and linear Schrödinger equation. Hence, most classical systems are improper for simulating qubit dynamics. In this article, we derive the conditions under which classical pendula with modulated coupling nevertheless can be described by a Schrödinger-like equation. We demonstrate this classical-to-quantum analogue by exploring three realizations of qubit control, namely Rabi oscillations^4^, LZ transitions^5,6^ and, finally, LZSM interferometry^7,8^.

Recent developments in quantum technology have motivated theoretical^1,9–14^ and experimental^15–18^ projects exploring analogues between classical coupled oscillators and its quantum version. The most interesting dynamics happens at avoided crossings of the eigenmodes of coupled oscillators near resonance. Previous theoretical considerations^10,12,13^ and experiments^17,18^ with nanomechanical oscillators used a time-dependent frequency difference corresponding to the detuning usually modulated in case of qubits^19–27^. To experimentally establish a classical-to-quantum analogue based on macroscopic pendula we instead modulate the coupling, which for this system is more practical than driving the detuning. This gimmick, for the first time allows us to continuously monitor the coherent dynamics of a driven two-level system at ambient conditions and to observe it with bare eyes. As we establish a one-to-one correspondence, our coupled pendula directly visualize the coherent dynamics of a driven qubit.

## Setup and model

**Figure 1:**
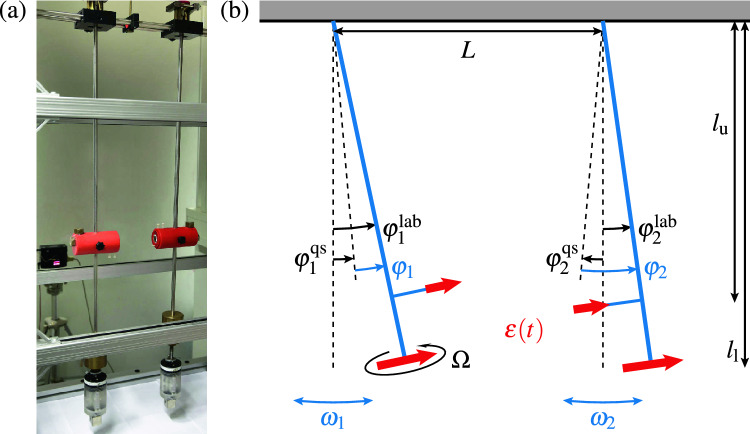
Photograph (**a**) and sketch (**b**) of the pendula coupled via cubic neodymium magnets (red arrows in sketch indicate magnetic moments), a lower pair with moments $m_\text {l}=25.37\,\text {Am}^2$ and an optional upper pair with $m_\text {u}=6.54\,\text {Am}^2$. The lower magnets are attached at the end of the pendula while the upper ones sit inside the red cylinders. Their respective distances from the pivots are $l_\text {l}=1.148\,$ m versus $l_\text {u}=0.635\,$ m. One of the lower magnets is slowly rotated at angular frequency $\Omega$ around the pendulum rod by a battery driven motor (inside the transparent plastic cases). The distances *L* between the pivots and $L_\text {u}$ between the upper magnets at deflections $\varphi ^\text {lab}_1=\varphi ^\text {lab}_2=0$ as well as $\Omega$ are variable. Each pendulum weighs 4.242 kg, where a 2.1 kg brass weight (visible in the photo) can be moved along a threaded section of each rod to vary $\omega _1$ and $\omega _2$.

In Fig. 1 we display a photograph and a simplified sketch of the setup (compare the attached movie Supplementary information). It consists of two pendula, each being described by its deflection angle $\varphi ^\text {lab}_k$ and its angular frequency $\omega _k$ with $k=1,2$. The two pendula are coupled via permanent magnets and detuned by the frequency difference $\Delta =\omega _1-\omega _2$. To probe the dynamics of qubits, usually the energy detuning between the diabatic states is modulated. However, modulating the coupling is mathematically equivalent after applying the appropriate basis transformation. For our system it is more practical to modulate the coupling. For this purpose we employ a battery driven linear motor, which rotates one of the magnets around the axis defined by the pendulum rod. As a result, the coupling and, at the same time, the equilibrium deflections of the pendula, $\varphi _k^\text {qs}$, are periodically modulated in time. The latter correspond to the quasistatic solution of the driven system, describing the (momentary) adiabatic equilibrium position.

We consider the deviation from the adiabatic equilibrium, $\varphi _k = \varphi ^\text {lab}_k - \varphi _k^\text {qs}$. Aiming at a description in the form of a Schrödinger equation, $i\partial _t\Psi =H\Psi$, which is linear and of first order, experiments and theory have to facilitate a linearization of the non-linear Newton EOM. This requires small deflection angles, small frequency differences and similar moments of inertia of the uncoupled pendula. The linearized version of the EOM reads

1 $$\begin{aligned} \begin{aligned} \ddot{\varphi }_1 + \omega _1^2\varphi _1 ={} \omega _0\varepsilon (t) (\varphi _1-\varphi _2), \\ \ddot{\varphi }_2 + \omega _2^2\varphi _2 ={} \omega _0\varepsilon (t) (\varphi _2-\varphi _1), \end{aligned} \end{aligned}$$

where $\omega _0=\frac{1}{2}(\omega _1+\omega _2)$ is the average pendulum (angular) frequency and $\varepsilon (t)$ is the coupling in units of frequency. (In the interaction term, we have neglected the small difference of the moments of interia. Moreover, the sign of $\varepsilon (t)$ is chosen such that it matches the usual definition in the quantum mechanical two-level problem. It is positive for attractive interaction.) The symmetry of the interaction terms on the right-hand side of Eq. 1 is essential for resembling the Schrödinger equation and requires neglecting the difference between the moments of inertia. Our modulated coupling, $\varepsilon (t)$, corresponds to the time-dependent level detuning commonly used to drive qubits, for instance in the context of the quantum mechanical LZSM problem^28^. To simplify a comparison with typical qubit experiments we aim at a coupling of the common form $\varepsilon (t) = \varepsilon _0 + A\cos (\Omega t)$, which renders Eq. 1 a Mathieu equation^29^. This modulation requires an experimental setup allowing for the far-field approximation of the dipole-dipole interaction.

For simulating qubit experiments we would like to independently modify the mean coupling $\varepsilon _0$ and the modulation amplitude *A*. To achieve this, we use two sets of magnet pairs, see Fig. 1. The lower magnets are attached at the distance $l_\text {l}$ from the pivots and the upper magnets at $l_\text {u}$, where $(l_\text {l}-l_\text {u})/L$ is sufficiently large to allow us to neglect quadrupole components of the coupling. The coupling is then composed of the sum of the contributions of the upper versus lower magnets, $\varepsilon =\varepsilon _\text {u}+\varepsilon _\text {l}$, where we slowly modulate $\varepsilon _\text {l}$ by rotating one of the lower magnets. The time-dependence of the reference point of the linearization, $\varphi _k^\text {qs}(t)$, leads to harmonic mixing such that $\varepsilon _0$ aquires a contribution from the rotating magnets and, vice versa, *A* is also affected by the static magnets.

The linearized EOM Eq. (1), which is still of second order, describes the free oscillations of two pendula with modulated coupling. In comparison, the Schrödinger equation of a qubit describes probability functions. These correspond to the slowly varying occupation amplitudes of the two pendula, given by the envelope functions, say $\Psi _k$, of the individual rapid oscillations $\varphi _k(t)$. To separate the time scales, we therefore employ the ansatz

2 $$\begin{aligned} \varphi _{k} = e^{-i\omega _0t} \Psi _{k} + \text {c.c.} \end{aligned}$$

with a rapidly oscillating prefactor and slowly varying complex envelopes $\Psi _{k}$. Inserting the ansatz into Eq. (1), while neglecting second order derivatives of $\Psi _k$, we find

3 $$\begin{aligned} i\frac{d}{dt}\begin{pmatrix} \Psi _1 \\ \Psi _2 \end{pmatrix} = \frac{1}{2}\begin{pmatrix} \Delta -\varepsilon (t) &{} \varepsilon (t) \\ \varepsilon (t) &{} -\Delta -\varepsilon (t) \end{pmatrix} \begin{pmatrix} \Psi _1 \\ \Psi _2 \end{pmatrix}, \end{aligned}$$

which for $\hbar =1$ possesses the form of a Schrödinger equation of the driven two-level system in the representation frequently found in textbooks for the Rabi problem (in a gauge without $\varepsilon$ in the diagonal).

For describing LZ transitions of a qubit with time-dependent detuning one usually uses the diabatic basis, in which the constant tunnel coupling appears in the off-diagonal matrix elements of the Hamiltonian. As we modulate the coupling between our pendula, it is convenient to transform into the according diabatic basis of the in-phase and out-of-phase modes, $\varphi _\pm = (\varphi _1\pm \varphi _2)/2$, in which the constant frequency difference $\Delta$ appears in the off-diagonal elements of the Hamiltonian. With $\Psi _\pm$ defined in accordance with Eq. (2) the presentation of the LZ problem for our pendula then reads

4 $$\begin{aligned} i\frac{d}{dt}\begin{pmatrix} \Psi _+ \\ \Psi _- \end{pmatrix} = \frac{1}{2} \begin{pmatrix} 0 &{} \Delta \\ \Delta &{} -2\varepsilon (t) \end{pmatrix} \begin{pmatrix} \Psi _+ \\ \Psi _- \end{pmatrix}. \end{aligned}$$

Equations (3) and (4) provide the foundation for comparing the dynamics of classical pendula with that of a qubit. They describe the occupation amplitudes of our coupled pendula in the form of a Schrödinger equation in the two alternative bases $\{\varphi _1,\varphi _2\}$ versus $\{\varphi _+,\varphi _-\}$. In Appendices A and B (Supplementary information) we offer an elegant alternative derivation based on the Lagrange formulation of classical mechanics and starting from the non-linear Newton equation. We also demonstrate there, how the time-dependent quasi static equilibrium, $\varphi ^\text {qs}_k(t)$, contributes to the static $\varepsilon _0$ and discuss limitations of our approximations.

In a nutshell, Eq. (3) describes —for the case of a time dependent coupling between the pendula— the dynamics of the occupation amplitudes of the individual pendula. In this way, the eigenmodes of the uncoupled pendula directly correspond to the wave functions of the localized sates of a qubit. Equation (3) is the natural choice for predicting Rabi oscillations occurring between the occupation amplitudes of the individual pendula for weak coupling. A basis transformation yields Eq. (4), which describes the dynamics between the occupation amplitudes of the in-phase and out-of-phase superposition modes of the individual pendula. Without driving, they resemble the eigenmodes of two strongly coupled pendula and correspond to the eigenfunctions of a qubit at zero detuning. Consequently, Eq. (4) is the natural choice for predicting the dynamics of the occupation amplitudes in the regime of LZ transitions, where the maximum coupling exceeds the detuning. See Table 1 and Appendix B3 (Supplementary information) for a one-to-one comparison between the parameters of a qubit and the pendula.

**Table 1:** Correspondence between a qubit described by the Schrödinger equation and the classical coupled pendula after the rotating-wave approximation, which alters the EOM independent of the carrier frequency $\omega _0 = (\omega _L+\omega _R)/2$.

Two-level system	Coupled pendula
Eigenstates	Normal modes
Tunnel oscillations	Beating
Tunnel coupling $\Delta$	Frequency diff. $\Delta = \omega _1-\omega _2$
Energy detuning $\varepsilon (t)$	Interaction $\varepsilon (t)$
Localized states	In-phase/out-of-phase mode
Delocalized states	Left/right pendulum mode
Amplitude of wavefunctions	Amplitude of pendula
Occupation probability	Occupation $\propto$ energy

Each line contains two corresponding quantities.

In our experiments we control *A* and $\varepsilon _0$ by varying the distance *L* between the lower magnets, corresponding to the distance between the pivots, and optionally the distance $L_\text {u}$ between the upper magnets [positioned inside the red horizontal cylindric housings seen in Fig. 1a], both defined for $\varphi _1=\varphi _2=0$. In addition, we adjust the frequency difference, $\Delta$, by moving a heavy weight [vertical brass cylinders partly visible in Fig. 1a] using a standard thread along one of the pendulum rods. We employ a line scan camera to simultaneously image at a rate of 10 Hz the lateral positions of both pendula within a linear pixel array. Applying numerical filtering we then obtain the displacement angles $\varphi _{k}(t)$ as a function of time, see Appendix E (Supplementary information). The mean frequency of our pendula is close to $\omega _0/2\pi \simeq 0.5$ Hz. To ensure the validity of the linearized Eq. (1), we work with small deflections $|\varphi _k|<1^\circ$, modulation frequencies $\Omega <10^{-2}\omega _0$ and frequency differences $|\Delta |<0.1 \omega _0$. Thereby $\omega _0$ is always the largest frequency by far supporting the separation of time scales in Eq. (2). The quality factor of $Q>2500$ of the coupled pendula is high enough to allow us ignoring dissipation as we did in the model above. In order to achieve high and stable quality factors, we employ professional pendulum clock pivots based on leaf springs provided by the company Erwin Sattler GmbH & Co. KG. In detail our experimental results indicate, that the damping of the coupled pendula motion is dominated by magnetic induction, i.e., eddy currents induced inside the conducting magnets due to their relative motion. The friction of air and the deforming pivot springs dominate the damping of the uncoupled pendula with stable quality factors of $Q>10000$. Note, that we can easily achieve the strong coupling regime as our maximal coupling of $|\varepsilon |_\text {max}\simeq 0.2$ Hz exceeds the resonance line width of $\omega _0/2\pi Q\sim 10^{-4}\,$ Hz by more than three orders of magnitude.

## Analysis and discussion

To explore the analogy between our coupled pendula and a qubit we first perform Rabi oscillations between the individual pendula in the limit of fast driving with $\Omega \simeq |\Delta |\gg A$, where the driving amplitude becomes the Rabi frequency, $\Omega _\text{ R }\simeq A$. After that, we turn to “qubit manipulation” using LZ transitions between the superposition modes, $\varphi _\pm$, in the limit of slow driving, $\Omega \ll |\Delta |<A$.

**Figure 2:**
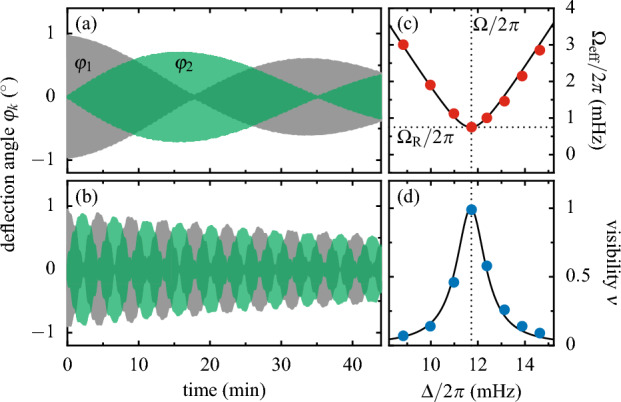
Near resonance Rabi oscillations between the two pendula with mean frequency $\omega _0/2\pi \simeq 528$ mHz, frequency difference $\Delta /2\pi =11.7\,$ mHz and modulation frequency $\Omega /2\pi =11.8\,$ mHz. At $t=0$ pendulum 1 was deflected at maximally attracting lower and no upper magnets. Individual oscillations are not visible owing to the time axis covering 45 minutes. (**a**, **b**) Deflections $\varphi _1(t)$ and $\varphi _2(t)$ of the two pendula for the pivot distances $L=496.5\,$ mm and $L=330.0\,$ mm resulting in Rabi frequencies of $\Omega _\text{ R }/2\pi =0.47\,$ mHz versus $\Omega _\text{ R }/2\pi =3.69\,$ mHz. (**c**, **d**) Effective frequency $\Omega _\text {eff}(\Delta )$ and visibility $\nu (\Delta )$ of the Rabi oscillations for $L=454.0\,$ mm. The solid lines represent model predictions.

In Fig. 2a,b we present Rabi oscillations for two different pivot distances *L* but otherwise identical conditions. We use no upper magnets and weak couplings (large *L*), such that $\varepsilon _0\ll A$ and $\varepsilon (t)\simeq A\cos (\Omega t)$. Shown are the deflections $\varphi _k(t)$ in respect to the equilibrium $\varphi _k^\text {qs}$. The observed beatings between the pendula are Rabi oscillations, where the variation between Rabi frequencies in Fig. 2a versus b reflect the differences in *L*. In both cases the energy transfer between the pendula is almost complete, as we chose a near resonance condition $\Omega \simeq |\Delta |$. Small steps, which occur at the repetition rate $2\Omega$ [zoom into Fig. 2a,b to clearly see them], indicate side band transitions beyond the rotating wave approximation. In Fig. 2b these steps are bigger due to a larger modulation amplitude compared to Fig. 2a.

By varying $\Delta$ we next explore the Rabi dynamics near resonance. In Fig. 2c we present the effective Rabi frequency $\Omega _\text {eff}(\Delta )$ corresponding to the actual beating frequency. Likewise, in Fig. 2d we show the visibility $\nu (\Delta )$ defined as the fraction of energy exchanged between the pendula. Symbols are measured data while the lines visualize the theory predictions, $\Omega _\text {eff}=[\Omega _\text{ R}^2+(\Omega -|\Delta |)^2]^{1/2}$ and $\nu =\Omega _\text{ R}^2/\Omega _\text {eff}^2$. The only fit parameter is the Rabi frequency $\Omega _\text{ R }/2\pi =0.71\,$ mHz, which defines the minimum of $\Omega _\text {eff}$ at resonance $\Omega =\Delta$ and which can be used to accurately determine the magnetic moment $m_\text {l}$, see Appendix C (Supplementary information). The excellent agreement between theory and experiment underlines the high quality of our classical mechanics experiment. Since the model curves can be derived from the Schrödinger equation (3), the result establishes a first analogue between classical pendula and a qubit.

**Figure 3:**
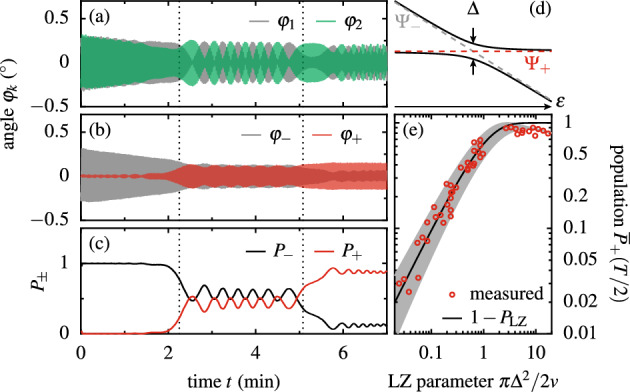
LZ transitions: At $t=0$ both pendula where deflected to excite the out-of-phase mode $\varphi _-$ at maximally attracting lower and no upper magnets. (**a**–**c**) Measured deflection angles $\varphi _{1,2}(t)$ (adiabatic modes) in a, diabatic modes $\varphi _\pm (t)$ in b and their occupations $P_\pm (t)$ in (**c**) during the first period $T=2\pi /\Omega$ of modulation for $\Omega /2\pi =2.27\,$ mHz, $\Delta /2\pi =6.7\,$ mHz, $\omega _0/2\pi =0.53$ Hz, and $P_\text {LZ}\simeq 0.4$. Two LZ transitions occur as the pendula pass through avoided crossings at $\varepsilon =0$ indicated by vertical dotted lines. The occupations $P_\pm (t)$ as well as the beating dynamics clearly change at each avoided crossing. (**d**) Sketch of the avoided crossing. Solid lines are the eigenfrequencies $(\mp \sqrt{\Delta ^2+\varepsilon ^2}-\varepsilon )/2$ and dashed lines indicate the frequencies of the envelope functions $\Psi _\pm (\varepsilon )$. (**e**) Mean population ${\overline{P}}_+(T/2)$ averaged over a proper time window around $t=T/2$ in between the first two crossings. Both, $\Delta$ and the LZ speed *v* are varied between individual measurements. The black line follows $1-P_\text {LZ}$. Our initialization at finite $\varepsilon (t=0)$ causes a small initial population of the upper mode $\varphi _+$, which varies from measurement to measurement in amplitude and phase. The gray region indicates the corrected range of prediction accounting for the range of experimental parameters by assuming arbitrary initial phases.

An elegant method to manipulate qubits in the limit of slow modulation, $|\Delta |\gg \Omega$, are LZ transitions^30,31^. In Fig. 3a we present the deflection angles $\varphi _{k}$, while one of the magnets completes one full rotation. Within this driving period, the pendula pass twice through the avoided crossing at zero coupling, sketched in Fig. 3d, namely from positive to negative $\varepsilon$ and back. At $t=0$, we initialized $\varphi _{1}=-\varphi _{2}$, such that $\varphi _+=0$. This is evident in Fig. 3b plotting the in-phase and out-of-phase combinations $\varphi _\pm =(\varphi _1\pm \varphi _2)/2$. In Fig. 3c we present the according populations $P_\pm$, given by the square modulus of the slowly varying amplitude, $P_\pm \propto |\Psi _\pm |^2$, normalized such that $P_++P_-=1$. Around the two avoided crossings (indicated by vertical dotted lines) we observe LZ transitions. The first one, which mixes $\varphi _+$ and $\varphi _-$, is followed by beats with the time dependent frequency $\sqrt{\Delta ^2+\varepsilon ^2(t)}$ clearly visible between $\varphi _{1}$ and $\varphi _{2}$ as well as between $P_-$ and $P_+$. The latter beats confirm theoretical predictions^19–21^, namely chirped oscillations centered around the LZ probability $P_\text {LZ} = \exp (-\pi \Delta ^2/2v)$ and $1-P_\text {LZ}$, respectively, right after passing through the avoided crossing. Here, $v = \Omega (A^2-\varepsilon _0^2)^{1/2}$ is the sweep velocity at $\varepsilon =0$, which depends on *L*, $L_\text {u}$ and $\Omega$. This observation demonstrates an advantage of our classical two-level system, which—in contrast to a qubit—allows us to trace the time evolution of population probabilities in real time in a single shot measurement. Based on the comparison with theory, we identify the long-time transition probability of a single LZ transition by averaging out the beats of the measured occupation $P_+(t)$ within an appropriate time window after half of the modulation period $T=2\pi /\Omega$, i.e., centered between the two LZ transitions. In Fig. 3e we compare the resulting ${{\overline{P}}}_+(T/2)$ for a wide range of the parameters $\Delta$, *L*, $L_\text {u}$ and $\Omega$ with the classic result $1-P_\text {LZ}$ for the limit $t\rightarrow \infty$^5–8^.

The agreement between model and measurements is good albeit compared to the Rabi experiment above the data scatter considerably around the model line. These deviations can be explained with the initialization into $\varphi _-$ at finite $\varepsilon$, which for $\Delta \ne 0$ is not an eigenmode, as it would be for an initialization at $\varepsilon \rightarrow -\infty$^5,6^. The weak admixture of the second eigenmode gives rise to a weak beating between $\varphi _\pm (t)$ right after initialization as visible in Fig. 3b for $t\lesssim 1\,$ min. Treating the relative phase between the modes $\varphi _\pm (t)$ (which could be predetermined at the cost of additional experimental effort) as an unknown, we predict the range of possible values of ${{\overline{P}}}_+(\pi /\Omega )$ for arbitrary phases [gray area in Fig. 3e]. In the adiabatic limit, $\Delta ^2/v\gg 1$, independent of the relative phase the finite occupation of the upper eigenmode, $P_+(t=0)>0$, results in ${{\overline{P}}}_+(\pi /\Omega )<1$ while $P_\text {LZ}=1$.

Note, that in a corresponding experiment with an actual qubit the initialization procedure would be similar, such that the phase problem described above occurs as well. However, only the classical qubit analogue allowed us to perform continuous measurements as those in Fig. 3c, which helped us to fully determine the influence of the non-zero phase at initialization. This result is an example of the usefulness of our classical approach for deciphering the sometimes complex dynamics of qubits.

**Figure 4:**
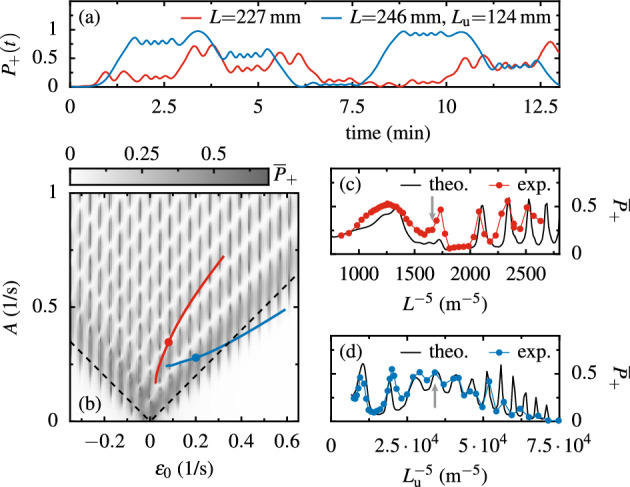
LZSM interference: (**a**) Measured occupation $P_+(t)$ of the in-phase mode $\varphi _+$, while one magnet was rotated at $\Omega ={7.1}~\hbox {mHz}$ with (blue) and without (red) static magnets. At $t=0$, $\varphi _-$ was excited as in Fig. 3. (**b**) Occupation $P_+$ averaged over the first 5 driving periods as a function of the average detunging $\epsilon _0$ and the effective driving amplitude *A*. The data are computed using Eq. (4). (**c**, **d**) Slices through the LZSM fan along the lines marked in panel b. Dots correspond to experimental results, while solid black lines show theory data from panel b. The colored dots in panel b and the arrows in panels c and d indicate the values used in panel a.

Each LZ transition mixes the eigenmodes as demonstrated in Fig. 3e. The resulting superposition state then accumulates the adiabatic phase $\int \text {d}t (\Delta ^2+\varepsilon (t)^2)^{1/2}$ integrating the difference between the two eigenfrequencies with the Stokes phase added^28^. The second LZ transition is then heavily influenced by these phases^7,8^. Indeed, multiple LZ transitions result in a complicated time evolution of $P_+(t)$ as can be seen in Fig. 4a, which presents two example time traces of $P_+(t)$. While the modulation frequency is identical for both measurements, $\Omega /2\pi =7.1\,$ mHz, we varied *A* and $\varepsilon _0$. Each trace covers five modulation periods corresponding to ten LZ transitions. Most of them are clearly visible as more or less pronounced steps while for some transitions $P_+(t)$ stays almost unchanged beyond the perpetual beating. The steady state solution for continuous driving averaging $P_+(t)$ over many periods gives rise to LZSM interference patterns ${{\overline{P}}}_+(A,\varepsilon _0)$, which can be used for exploring qubit dynamics, decoherence or multi-color driving^25,26^. For a practical comparison, allowing for small deviations, we average $P_+(t)$ over the initial five modulation periods. In Fig. 4b we present the LZSM interference pattern ${{\overline{P}}}_+(A,\varepsilon _0)$ as a gray scale, which we computed using the Schrödinger equation (4). In Fig. 4c,d we finally present interference traces along the two solid lines in Fig. 4b, mind the color code. In Fig. 4c we plot $P_+(L^5)$ for the case without upper magnets, while for Fig. 4d we added the upper magnets and show $P_+(L_\text {u}^5)$ for a constant pivot distance, $L=246$ mm. Solid lines are model curves calculated with Eq. (4) [contained in the gray scale plot in Fig. 4b], while the dots present measured interference patterns. Hereby, each point corresponds to the average of a $P_+(t)$ trace as those shown in Fig. 4a. Our measurements qualitatively reproduce the calculated interference fringes. Quantitative deviations indicate the limitations of the mapping of the Newton equation onto the Schrödinger equation, in particular for large *A* and $\epsilon _0$. In Appendix D (Supplementary information) we provide related background information.

## Summary

Wave mechanics introduced by Erwin Schrödinger in 1926^32^ provides a mathematical description for the coherent dynamics of a qubit. However, a continuous experimental visualization of its time evolution is hindered by the principle of projection measurement; each measurement would destroy the quantum coherence. In comparison, our classical qubit analogue surely allows to trace the complete time evolution in a single measurement. In turn, the collapse of the wave function cannot be simulated in our classical system. Another limit of macroscopic mechanical systems as ours is imposed by entanglement of multiple qubits, which has no classical analogue. Interestingly, using distinct degrees of freedom in classical optical beams this restriction has been circumnavigated^33–35^.

In order to actually visualize Schrödingers wave mechanics using physical pendula with modulated coupling one has to map the non-linear, second order and inhomogeneous classical EOM to the linear, first order and homogeneous Schrödinger equation. This mapping, which includes a linearization, a rotating wave approximation and a time-dependent shift of the reference point, also clarifies the experimental conditions necessary for classical qubit simulator experiments and their physical interpretations. In this spirit, we presented three key qubit experiments with coupled pendula, namely Rabi oscillations, LZ transitions and LZSM interferometry. Comparing measurements with the prediction of the Schrödinger equation we demonstrated that our classical experiments directly visualize Schrödinger’s wave mechanics.

Our classical qubit simulator bridges the gap between the elusiveness of quantum mechanics and the common imagination pre-shaped by classical experiences. The experimental setup is highly versatile and might be used for exploring a variety of phenomena beyond simulating a qubit, such as geometrical phases^36^, after adding more pendula, multi-level systems^37,38^ or coherent transfer by adiabatic passage^39^ or simulating the non-linear Schrödinger equation^40^. Moreover, driven systems of coupled pendula may serve as visualizer of a large variety of coupled systems in nature or even economical, social or financial systems.

### Supplementary Information

Supplementary Information 1.

Supplementary Information 2.

## Data Availability

The data generated during the current study are available from the corresponding author on reasonable request.
