# Publisher Correction: Classical analogue to driven quantum bits based on macroscopic pendula

**DOI:** 10.1038/s41598-023-46681-0

**Published:** 2023-11-07

**Authors:** Heribert Lorenz, Sigmund Kohler, Anton Parafilo, Mikhail Kiselev, Stefan Ludwig

**Affiliations:** 1 Fakultät für Physik, Center for NanoScience (CeNS), Ludwig-Maximilians-Universität (LMU), 80539 München, Germany; 2 Instituto de Ciencia de Materiales de Madrid, CSIC, 28049 Madrid, Spain; 3 Center for Theoretical Physics of Complex Systems (PCS), Institute for Basic Science (IBS), Expo-ro 55, Yuseong-gu, Daejeon, 34126 Korea; 4 The Abdus Salam International Centre for Theoretical Physics (ICTP), Strada Costiera 11, 34151 Trieste, Italy; 5 Paul-Drude-Institut für Festkörperelektronik (PDI), Leibniz-Institut im Forschungsverbund Berlin e.V., Hausvogteiplatz 5-7, 10117 Berlin, Germany

Correction to: *Scientific Reports* 10.1038/s41598-023-45118-y, published online 26 October 2023

The original version of this Article contained errors in Figure 2 where the gray data curves were incorrectly captured in panels (a) and (b).

The original Figure 2 and accompanying legend appear below.

**Figure 2:**
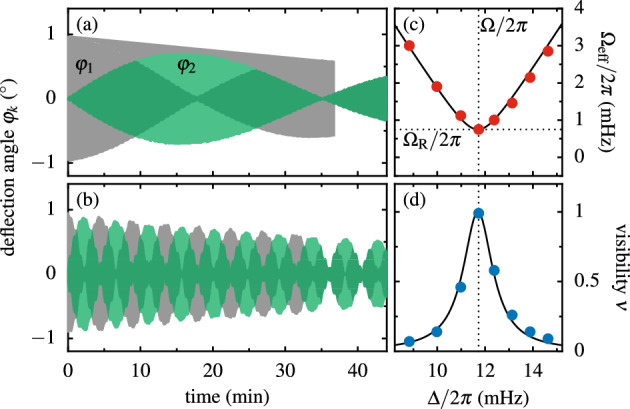
Near resonance Rabi oscillations between the two pendula with mean frequency $\omega _0/2\pi \simeq 528$ mHz, frequency difference $\Delta /2\pi =11.7\,$ mHz and modulation frequency $\Omega /2\pi =11.8\,$ mHz. At $t=0$ pendulum 1 was deflected at maximally attracting lower and no upper magnets. Individual oscillations are not visible owing to the time axis covering 45 minutes. (**a**, **b**) Deflections $\varphi _1(t)$ and $\varphi _2(t)$ of the two pendula for the pivot distances $L=496.5\,$ mm and $L=330.0\,$ mm resulting in Rabi frequencies of $\Omega _\text{ R }/2\pi =0.47\,$ mHz versus $\Omega _\text{ R }/2\pi =3.69\,$ mHz. (**c**, **d**) Effective frequency $\Omega _\text {eff}(\Delta )$ and visibility $\nu (\Delta )$ of the Rabi oscillations for $L=454.0\,$ mm. The solid lines represent model predictions.

The original Article has been corrected.

