# Supplementary material for: Classical analogue to driven quantum bits based on macroscopic pendula
Source: Sci Rep. 2023 Oct 26;13:18386. doi: 10.1038/s41598-023-45118-y (PMC10603110; doi:10.1038/s41598-023-45118-y)
Supplement: Supplementary file 2 — Supplementary Information 2. [file 41598_2023_45118_MOESM2_ESM.pdf]

Classical Analogue to Driven Quantum Bits based on Macroscopic Pendula

Heribert Lorenz,[1] Sigmund Kohler,[2] Anton Parafilo,[3] Mikhail Kiselev,[4] and Stefan Ludwig[5]
[1]Center for NanoScience (CeNS) & Fakultät für Physik, Ludwig-Maximilians-Universität (LMU), 80539 München, Germany
[2]Instituto de Ciencia de Materiales de Madrid, CSIC, 28049 Madrid, Spain
[3]Center for Theoretical Physics of Complex Systems (PCS), Institute for Basic Science (IBS), Expo-ro 55, Yuseong-gu, Daejeon 34126, Korea
[4]The Abdus Salam International Centre for Theoretical Physics (ICTP), Strada Costiera 11, 34151 Trieste, Italy
[5]Paul-Drude-Institut für Festkörperelektronik (PDI), Leibniz-Institut im Forschungsverbund Berlin e.V., Hausvogteiplatz 5-7, 10117 Berlin, Germany
(Dated: September 20, 2023)

Appendix A: Newton equation

To derive the equation of motion, we employ the Lagrange formalism. For two uncoupled physical pendula the Langrangian reads [1]

$$\mathscr{L}_k = \frac{1}{2} J_k \dot{\varphi}_k^2 + J_k \omega_k^2 \cos\varphi_k, \tag{A1}$$

where $k = 1,2$ labels the two so-far uncoupled pendula and the deflection angles φ_k are zero for vertically hanging pendula described by their moments of inertia J_k. In the second term, which is the negative potential energy, we have already made use of the fact that the eigenfrequency reads $\omega_k^2 = gM(l_\mathrm{c})_k/J_k$, with the gravity acceleration g, the mass M, which is equal for both pendula, and the distance $(l_\mathrm{c})_k$ between the pivot and the center of mass of each pendulum. Note, that up to Eq. (A15), φ_k denotes the deflection angles in the lab frame with the vertical direction as reference.

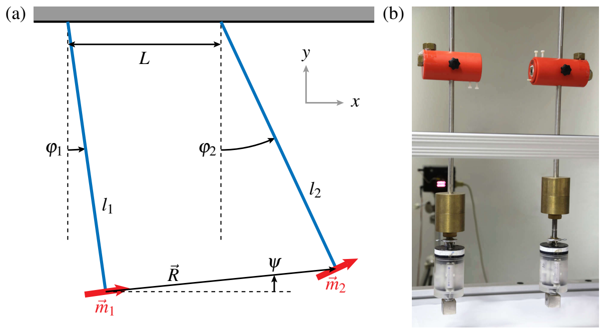

Figure S1. Sketch (a) and photograph (b) of the pendula, which are coupled by cubic neodymium magnets. The sketch visualizes the geometry of the pendula, the orientations of the magnets as well as the angles and lengths used in the derivation of the interaction potential. The two lower magnets with magnetizations $\vec{m}_1$ and $\vec{m}_2$ (with $|\vec{m}_1| = |\vec{m}_2|$) are attached at the end of the pendula to motors, which are encapsulated together with batteries and a circuit board by half transparent polethylene cylinders.The upper magnets, which have an approximately four times smaller magnetization, can be horizontally moved within the red ceramic cylinders in order to adjust their distance independently from the distance L between the pivots. With their magnetizations put parallel and in line they attract each other. Brass nuts attached to the cylinders have the purpose of balancing the weights of the magnets such that the center of masses stay within the pendulum rods. The 2.1 kg heavy brass weights above the motors can be vertically moved to tune the pendula's eigenfrequencies. The line scan camera in the background stroboscopically monitors the horizontal position of both pendula as a function of time using narrow reflectors (not seen).

1. Coupling by magnets

The two pendula are coupled by either one or two pairs of permanent neodymium magnets as sketched in Fig. S1(a) for the case of one pair of magnets. The cubical magnets can be seen in the photograph in Fig. S1(b). Two large magnets with 28 mm edge lengths are fixed to the end of each rod and can be rotated by battery driven motors. Two smaller magnets are positioned inside the red cylinders 0.513 m above. All magnet cubes are magnetized along their horizontal four-fold axes, which at all times remain fixed within the oscillation plane with the excumption of one of the lower magnets optionally rotated around the rod it is attached to.

With the origin set to the pivot of the left pendulum ($k = 1$), the magnets have positions

$$\vec{r}_1 = \begin{pmatrix} l\sin\varphi_1 \\ -l\cos\varphi_1 \\ 0 \end{pmatrix}, \quad \vec{r}_2 = \begin{pmatrix} L + l\sin\varphi_2 \\ -l\cos\varphi_2 \\ 0 \end{pmatrix}, \tag{A2}$$

where L is the horizontal distance between the pivots of the pendula, and l is the distance between magnet and pivot, which is equal for both pendula. Their magnetic dipole moments of identical magnitude m are oriented as

$$\vec{m}_1 = m \begin{pmatrix} \cos\varphi_1 \\ \sin\varphi_1 \\ 0 \end{pmatrix}, \quad \vec{m}_2 = m \begin{pmatrix} \cos\varphi_2 \cos(\Omega t) \\ \sin\varphi_2 \cos(\Omega t) \\ \sin(\Omega t) \end{pmatrix}, \tag{A3}$$

valid for large enough L as is the case in all our experiments.

The magnetic dipole-dipole coupling energy is [2]

$$\mathscr{U}_{12} = \frac{\mu_0}{4\pi R^3} \left[\vec{m}_1 \cdot \vec{m}_2 - 3(\vec{m}_1 \cdot \vec{e}_R)(\vec{m}_2 \cdot \vec{e}_R) \right], \tag{A4}$$

where $R\vec{e}_R = \vec{R} \equiv \vec{r}_2 - \vec{r}_1$. To express this in terms of our dynamical variables φ_k, we insert the vectors in Eqs. (A2) and (A3) and obtain the potential energy of the coupling to read $\mathscr{U}_{12} = \mathscr{V}_{12}\cos(\Omega t)$, where

$$\mathscr{V}_{12} = \frac{\mu_0 m^2}{4\pi R^3} \left[\cos(\varphi_1 - \varphi_2) - 3\cos(\varphi_1 - \psi)\cos(\varphi_2 - \psi) \right]. \tag{A5}$$

Length and orientation of $\vec{R}$ follow from straightforward geometric considerations and read

$$\begin{aligned} R &= \sqrt{(L + l\sin\varphi_2 - l\sin\varphi_1)^2 + (l\cos\varphi_2 - l\cos\varphi_1)^2}, \\ \psi &= \arctan\left(\frac{\cos\varphi_1 - \cos\varphi_2}{L/l - \sin\varphi_1 + \sin\varphi_2}\right), \end{aligned} \quad \text{(A6)}$$

where ψ is the angle between the x-axis and $\vec{R}$, cf. Fig. S1. In some of our experiments, we use two pairs of magnets, i.e., a lower pair connected to the ends of the rods and an upper pair with a variable horizontal distance shifted upwards along the rods. Consequently, we introduce the respective distances l_l (replacing l) and l_u between pivots and lower versus upper magnets. Hereby, $l_\mathrm{l} - l_\mathrm{u}$ exceeds the horizontal distance L roughly by a factor two, such that we can neglect the interaction between the upper and the lower magnets and consider two separate dipole-dipole couplings. The upper magnets orientations are fixed, such that their magnetizations are perpendicular to the respective rods, remain in the x-y plane and attract each other. In contrast, one of the lower magnets is rotated such that

$$\mathscr{U}_{12} = \mathscr{V}_{12}^{\mathrm{u}} + \mathscr{V}_{12}^{\mathrm{l}}\cos(\Omega t). \quad \text{(A7)}$$

The equations of motion follow readily from the Lagrange equation [1]

$$\frac{d}{dt}\frac{\partial \mathscr{L}}{\partial \dot{\varphi}_k} = \frac{\partial \mathscr{L}}{\partial \varphi_k}. \quad \text{(A8)}$$

For the full Lagrangian, $\mathscr{L} = \mathscr{L}_1 + \mathscr{L}_2 - \mathscr{U}_{12}$, they become

$$J_k\ddot{\varphi}_k + J_k\omega_k^2\sin\varphi_k = -\frac{\partial \mathscr{U}_{12}}{\partial \varphi_k}, \quad k = 1,2. \quad \text{(A9)}$$

The derivative of the interaction potential is straightforward to calculate but results in a bulky expression, whose explicit form is not needed for our discussion. Equations (A9) can be integrated numerically to obtain a full solution of the pendula dynamics which considers all nonlinearities.

2. Linearization and quasistatic solution

The mapping of Newton's equation of motion to a Schrödinger equation requires a bilinear Lagrangian, as will become clear in Sec. B below. In our experiment, the deflection angles are rather small, $|\varphi_k| \lesssim 1^\circ$, such that already a linearized version of the equation of motion provides a faithful description. We will perform this linearization by an expansion for the Lagrangian up to second order in φ_k. Then the Lagrangian of the uncoupled pendula, Eq. (A1), takes the bilinear form $\mathscr{L}_k = J_k(\dot{\varphi}_k^2 - \omega_k^2\varphi^2)/2$, where we have omitted an irrelevant constant term. The expansion of the interaction term (A5) requires more effort, because the φ_k appear in both the numerator and the denominator.

Closer inspection of the expressions, however, reveals that the φ_k-dependence of the numerator is much weaker than that of the denominator, given by $R^{-3}(\varphi_k)$, see Eq. (A6). The difference stems from the numerical pre-factors of the second-order terms of the Taylor expansions of $(1-x)^{-3}$ and $\cos(x)$, which are 12 and $1/2$, respectively. Moreover, in R the φ_k are weighted by a factor l/L, which for our experiment is ~ 4. This leads to the conclusion that the numerator is responsible for only $\sim 1\%$ of the curvature of $\mathscr{U}_{12}$. Hence, we neglect the higher order terms of the numerator and approximate $\cos(\varphi_1 - \varphi_2) - 3\cos(\varphi_1 - \psi)\cos(\varphi_2 - \psi) \simeq -2$. However, we do consider the terms stemming from the expansion of the denominator,

$$\begin{aligned} R^{-3} &= [L - l\varphi_1 + l\varphi_2 + \mathscr{O}(\varphi^4)]^{-3} \\ &\simeq L^{-3}\left(1 + 3\alpha + 6\alpha^2 + 10\alpha^3\right) \end{aligned} \quad \text{(A10)}$$

with $\alpha = (\varphi_1 - \varphi_2)l/L$. Inserting the relevant terms up to second order into Eq. (A5) yields the Langrangian of the linearized problem

$$\mathscr{L} = \sum_{k=1,2}\frac{J_k}{2}(\dot{\varphi}_k^2 - \omega_k^2\varphi_k^2) + \tilde{F}(\varphi_1 - \varphi_2) + \frac{\tilde{G}}{2}(\varphi_1 - \varphi_2)^2. \quad \text{(A11)}$$

For our two pairs of magnets, where one of the lower magnets is rotating, the energies $\tilde{G}$ and $\tilde{F}$ take the form

$$\begin{aligned} \tilde{G}(t) &= G_\mathrm{u} + G_\mathrm{l}\cos(\Omega t), \\ \tilde{F}(t) &= \frac{L_\mathrm{u}}{4l_\mathrm{u}}G_\mathrm{u} + \frac{L}{4l_\mathrm{l}}G_\mathrm{l}\cos(\Omega t), \end{aligned} \quad \text{(A12)}$$

where the tilde indicates parameters stemming from second-order Taylor expansion of the Lagrangian. Equation (A12) neglects the time-dependent equilibrium positions of the pendula, which we will take into account as a correction below. The coefficients in Eq. (A12), which quantify the interaction energies are determined by $\mathscr{U}_{12}$ and read

$$\begin{aligned} G_\mathrm{u} &= \frac{6\mu_0 m_\mathrm{u}^2 l_\mathrm{u}^2}{\pi L_\mathrm{u}^5}, \\ G_\mathrm{l} &= \frac{6\mu_0 m_\mathrm{l}^2 l_\mathrm{l}^2}{\pi L^5}, \end{aligned} \quad \text{(A13)}$$

where L_u is the distance between the upper magnets at $\varphi_1 = \varphi_2 = 0$. L_u is varied between experiments and $L_\mathrm{u} = \infty$ corresponds to the case without upper magnets.

To bring $\mathscr{L}$ into a bilinear form, we transform the coordinates such that the linear term $\tilde{F}(\varphi_1 - \varphi_2)$ on the right-hand side of Eq. (A11) vanishes. This can be achieved by introducing coordinates relative to the potential minimum which is located at

$$\begin{aligned} \varphi_1^{\mathrm{qs}}(t) &= \frac{J_2\omega_2^2\tilde{F}(t)}{J_1J_2\omega_1^2\omega_2^2 - \tilde{G}(t)(J_1\omega_1^2 + J_2\omega_2^2)}, \\ \varphi_2^{\mathrm{qs}}(t) &= -\frac{J_1\omega_1^2\tilde{F}(t)}{J_1J_2\omega_1^2\omega_2^2 - \tilde{G}(t)(J_1\omega_1^2 + J_2\omega_2^2)}, \end{aligned} \quad \text{(A14)}$$

where the superscript "qs" refers to the quasistatic solution. The adiabatic equilibrium positions φ_k^{qs} implicitly depend on the center-of-mass coordinates since $J_k\omega_k^2 = Mg(l_\mathrm{c})_k$.

Our driving frequency is much smaller than the resonance frequencies of the two pendula, $\Omega/\omega_0 \lesssim 0.01$, where $\omega_0 = (\omega_1+\omega_2)/2$. Hence, within an adiabatic approximation, Eq. (A14) describes the steady-state solution of the coupled pendula driven by the rotation of one of the magnets. Henceforth, we use $\varphi_k^{\rm qs}(t)$ as reference point, which is achieved by the transformation

$$\varphi_k \to \varphi_k^{\rm lab} = \varphi_k + \varphi_k^{\rm qs}(t)\,. \tag{A15}$$

Moreover, we neglect within an adiabatic approximation the time derivatives of $\varphi_k^{\rm qs}(t)$ which removes the linear term in Eq. (A11) and results in the desired intended bilinear Lagrangian.

By applying the transformation (A15) the corresponding equation of motion becomes not only of first order and linear, but also homogeneous. Accordingly, it assumes the form of a Schrödinger equation. However, the coordinates φ_k are no longer in the lab frame. Instead, they are the deflection angles with respect to the time-dependent quasistatic solution in Eq. (A14).

3. Dynamic potential curvature

The price for separating φ_k from the time dependence of the equilibrium positions of the pendula is that for the new coordinates the interaction becomes time dependent even for constant φ_1 and φ_2. In a hand-waving picture, while one magnet is rotating the equilibrium distance between the magnets is smaller whenever the interaction is attractive as compared to the case of repulsive interaction. As a result, the interaction itself is accordingly modulated for any values of φ_1 and φ_2. We will now quantify this correction.

So far, we have linearized the equation of motion by performing a Taylor expansion of the interaction potential at the pivot distance L. However, after the transformation (A15), the potential curvature at $\varphi_1 = \varphi_2$ is no longer given by the energy $\tilde{G}(t)$ in Eq. (A12), but by the corresponding Taylor coefficient evaluated at $L - l_{\rm u}\delta\varphi^{\rm qs}(t)$ for $G_{\rm u}$ and $L - l_{\rm l}\delta\varphi^{\rm qs}(t)$ for $G_{\rm l}$. This can be captured by the approximate correction

$$\begin{aligned}\tilde{G}(t) \to G(t) = {}& G_{\rm u}\Big[1 - \frac{l_{\rm u}}{L_{\rm u}}\delta\varphi^{\rm qs}(t)\Big]^{-5} \\ & + G_{\rm l}\cos(\Omega t)\Big[1 - \frac{l_{\rm l}}{L}\delta\varphi^{\rm qs}(t)\Big]^{-5}.\end{aligned} \tag{A16}$$

Clearly, the interaction term acquires a further time dependence via $\delta\varphi^{\rm qs}(t) \equiv \varphi_1^{\rm qs}(t) - \varphi_2^{\rm qs}(t)$ in addition to the modulation expressed in Eq. (A12). The explicit dependence of $G(t)$ on $\delta\varphi^{\rm qs}(t)$ results in an asymmetry between the times of attractive versus repulsive magnetic force even without upper magnets.

To see the consequences of this correction, we restrict ourselves to weak asymmetries such that the pendulum frequencies and moments of inertia can be replaced by their average values ω_0 and $J_0 = (J_1+J_2)/2$. Then we arrive at the approximation

$$\varphi_1^{\rm qs}(t) = \frac{\tilde{F}(t)}{\omega_0^2 J_0 - 2\tilde{G}(t)} = -\varphi_2^{\rm qs}(t), \tag{A17}$$

which allows the numerical evaluation of the effective potential curvatures using Eq. (A16).

To make analytical progress, we restrict ourselves to the case in which only the rotating lower magnets are present, while $G_{\rm u} = 0 = F_{\rm u}$. Our aim is to show that, in consistency with the experimental observation, nevertheless the effective potential curvature has a non-vanishing mean value. In doing so, we keep only corrections to lowest order in $G(t)$ and, hence, in $\delta\varphi^{\rm qs}(t)$ such that the effective potential curvature becomes

$$\begin{aligned} G(t) &\simeq G_{\rm l}\cos(\Omega t)\Big[1 + \frac{5}{2}\frac{G_{\rm l}\cos(\Omega t)}{\omega_0^2 J_0}\Big] \\ &= G_{\rm l}\cos(\Omega t) + \frac{5G_{\rm l}^2}{4\omega_0^2 J_0}[1+\cos(2\Omega t)].\end{aligned} \tag{A18}$$

The first term describes the modulation of the interaction by the rotating magnet in accordance to Eq. (A12). The second constant term describes the average increase of the interaction related to the fact that the dipole-dipole interaction is more enhanced during attraction than reduced during repulsion. The third term describes a second harmonic modulation in Ω. The latter merely distorts the shape of the driving and can be neglected on the level of agreement we are aiming at.

Let us emphasize that the derivation of the correction (A16) relies on the assumption that the nonlinear φ_k dependence of the interaction $\mathscr{U}_{12}$ can be captured by a time-dependent quadratic term. While this reasoning naturally has limitations, it clearly reveals that the varying potential curvature causes an effective constant interaction term, even in the absence of the upper magnets. It explains the observed dependences $\varepsilon_0(L)$ and $A(L)$ without upper magnets, cf. the red line in Fig. 4b of the main article or Fig. S7 below.

Appendix B: Schrödinger-like equation

Next, we bring the linearized equation of motion of our system to the form of a Schrödinger equation that describes the time-dependent amplitude of the fast oscillations of each pendulum with approximately the average frequency $\omega_0 = (\omega_1 + \omega_2)/2$. The pedestrians way [3–5] starts from the linearized classical equation of motion which is of second order. To obtain a differential equation of first order, one employs for the deflection angles the complex ansatz $\varphi_k = \Re e^{-i\omega_0 t}\Psi_k + \text{c.c.}$, $k = 1,2$, where Ψ_k is a slowly varying amplitude. Within a rotating-wave approximation, one then neglects the second-order derivatives of Ψ_k and all terms that oscillate with the angular frequency ω_0.

Here we pursue a more elegant alternative by performing these steps within the Lagrange formalism. Accordingly, our goal is to transform Eq. (A11) such that it assumes the form of the Lagrangian of the Schrödinger equation $i\hbar\partial_t\Psi = H\Psi$, which reads

$$\mathscr{L}_{\rm Schr} = i\hbar\Psi^\dagger\partial_t\Psi - \Psi^\dagger H\Psi, \tag{B1}$$

where Ψ and H denote the probability amplitudes and the Hamiltonian in vector-matrix notation. Alternatively, one

may use the symmetrized form of the Lagrangian, $\mathscr{L}_{\rm Schr} = i\hbar(\Psi^\dagger\partial_t\Psi - \Psi\partial_t\Psi^\dagger)/2 - \Psi^\dagger H\Psi$, which differs from Eq. (B1) by an irrelevant total time derivative. Its relation to the Schrödinger equation follows readily from the Langrange equation

$$\frac{d}{dt}\frac{\partial\mathscr{L}}{\partial\dot{\Psi}_k^*} = \frac{\partial\mathscr{L}}{\partial\Psi_k^*}. \tag{B2}$$

The fact that $\mathscr{L}_{\rm Schr}$ is bilinear, makes it obvious that it was indispensable to remove the term of Eq. (A11) linear in φ_k via the transformation (A15) and to avoid explicit terms of higher order.

Let us stress that the resulting equation still describes classical mechanics and, despite its form, does not constitute a quantization. In particular, the quantum mechanical energy-frequency relation given by Planck's constant does not hold. Technically, this is not a problem as long as we use a Schrödinger equation in the dimensions of frequency. If we assume $\hbar = 1$, it is identical to the usual quantum version of the Schrödinger equation in units of frequency.

1. Hamiltonian and conservation law

Like in the standard approach, we assume that the average pendulum frequency ω_0 is much larger than all other frequency scales, which suggests an ansatz with a rapid oscillation and a slowly varying amplitude. To be specific, we define

$$\varphi_k(t) = \Psi_k(t)e^{-i\omega_0 t} + \text{c.c.}, \tag{B3}$$

where Ψ_k is generally complex. While inserting this ansatz into Eq. (A11), we keep only terms that are at least of order ω_0. (The ansatz is constructed such that terms $\propto \omega_0^2$ cancel each other.) Moreover, we neglect within a rotating-wave approximation all terms that contain the phase factor $e^{\pm i\omega_0 t}$.

In the resulting expression, the remaining part of the first term of $\mathscr{L}$ in Eq. (A11) becomes $2i\omega_0 J_k\Psi_k\partial_t\Psi_k$. It is still not of the desired form, because its pre-factor still depends via the moment of inertia on the mode index k. To reach the form of a Schrödinger equation, one needs pre-factors independent of the mode index k, e.g., by re-scaling the amplitude with a factor $J_k^{-1/2}$. This however would no longer allow the intuitive interpretation of the $\Psi_\pm$ defined below as in-phase and out-of-phase modes. To nevertheless get rid of the k-dependence of the pre-factors, we replace the J_k by their average $J_0 = (J_1 + J_2)/2$. This is a reasonable approximation for most of our experiments, as they fulfill $|J_1 - J_2| \ll (J_1 + J_2)$. Finally, for convenience we divide by $2\omega_0 J_0$ (which has no consequence for the equations of motion) to obtain a Lagrangian with dimension frequency

$$\mathscr{L} = i\sum_k \Psi_k^*\dot{\Psi}_k - \frac{\Delta}{2}(|\Psi_1|^2 - |\Psi_2|^2) + \frac{\varepsilon}{2}|\Psi_1 - \Psi_2|^2, \tag{B4}$$

where we have introduced the frequency difference

$$\Delta = \omega_1 - \omega_2 \tag{B5}$$

and the time dependent coupling (in units of frequency)

$$\varepsilon(t) = \frac{G(t)}{\omega_0 J_0}. \tag{B6}$$

Comparison with Eq. (B1) demonstrates that the Lagrangian in Eq. (B4) corresponds to a Schrödinger equation of a two-level-system (TLS) with the Hamiltonian

$$H = \frac{1}{2}\begin{pmatrix} \Delta - \varepsilon(t) & \varepsilon(t) \\ \varepsilon(t) & -\Delta - \varepsilon(t) \end{pmatrix} \tag{B7}$$

in units of frequency with $\hbar = 1$.

The $U(1)$ symmetry of the Lagrangian (B4) together with Noether's theorem immediately provides the conservation of $N \equiv |\Psi_+|^2 + |\Psi_-|^2$. Then the energy $\mathscr{E} = \mathscr{T} + \mathscr{U}$ of the pendula obeys the proportionality

$$\mathscr{E} = \omega_0^2 N + 2\omega_0\langle H\rangle. \tag{B8}$$

It is dominated by the constant term $\omega_0^2 N$, while the expectation value $\langle H\rangle$ represents a small, generally time-dependent correction to the energy of the driven coupled pendula. Because of the driving, normalization of the wave function corresponds to a merely approximate energy conservation of the pendula motion.

2. Basis transformation

The preferential basis in most works on quantum dots is the one formed by localized states. Then ac voltages applied via plunger gates appear in the Hamiltonian as time-dependent diagonal elements. To establish a closer connection to these systems, we introduce the in-phase and out-of-phase mode $\varphi_\pm = \varphi_1 \pm \varphi_2$ and the corresponding envelopes $\Psi\pm = \Psi_1 \pm \Psi_2$. This corresponds to a unitary transformation with $S = (\sigma_x + \sigma_z)/\sqrt{2}$, which formally interchanges the Pauli matrices σ_x and σ_z. With $H \to SHS^\dagger$ our Schrödinger equation takes the form

$$i\frac{d}{dt}\begin{pmatrix}\Psi_+ \\ \Psi_-\end{pmatrix} = \begin{pmatrix} 0 & \Delta/2 \\ \Delta/2 & -\varepsilon(t)\end{pmatrix}\begin{pmatrix}\Psi_+ \\ \Psi_-\end{pmatrix}, \tag{B9}$$

which represents the basis of the analogy between the pendula and the quantum mechanical two-level system.

3. Duality and limitations

We have chosen the diabatic basis spanned by the in-phase and out-of-phase modes. The corresponding delocalized basis for the Hamiltonian in Eq. (B9) corresponds to a localized basis usually employed for LZSM physics with a qubit, e.g., the one defined by the localized electron states in a double quantum dot [6]. In Table I in the main article we present the cross relation between the localized and delocalized bases corresponding to mapping our coupled pendula to a double dot qubit.

An interesting point arises from the fact that we modulate the coupling between the pendula while in qubits usually the detuning between localized states is modulated. The consequence is a duality between the terms "detuning" and "coupling" in Table I in the main article: The coupling of the pendula by the magnetic interaction corresponds to the detuning of the quantum dot levels, while the detuning of the pendulum frequencies corresponds to the tunnel coupling.

The mapping to a Schrödinger equation relies on the slowly-varying envelope approximation which requires $\Delta, \varepsilon \ll \omega_0$. While usually the case, for our smallest pivot distance, $L = 208\,\mathrm{nm}$, this is fulfilled only marginally as we find $|\varepsilon|$-values up to $1\,\mathrm{s}^{-1}$ while $\omega_0 \simeq 3.3\,\mathrm{s}^{-1}$. Indeed, such large $|\varepsilon|$-values already significantly reduce the potential curvatures in some direction such that with increasing coupling, one eigenmode eventually becomes unstable. This happens when for $\varepsilon(t) > \omega_0$ the adiabatic eigenfrequencies of the linearized Newton equation become imaginary during some time intervals, see Fig. S3 below and its discussion in Sec. C 3.

Finally, we comment on the approximation by which we replaced the moments of interia J_1 and J_2 by their average, i.e., we neglected terms propotional to $\delta J = J_1 - J_2$. If we had kept this term and employed the ansatz (B3), our Hamiltonian would have acquired a non Hermitian contribution. Its size might be reduced by a more sophisticated ansatz, but then the modes would depend on the J_k and, thus, on the parameter sets. For our LZSM-interferometry experiments, $\delta J \ll J_{1,2}$, such that practical corrections are minor. In the case of our Rabi experiments $\delta J \leq 0.2 J_{1,2}$, which is sufficient for the weak couplings considered there.

Appendix C: Driven qubit dynamics

So far, we have shown that within the range of validity of the rotating-wave approximation, the oscillations of our coupled pendula have an envelope which obeys the Schrödinger equation of the TLS. Next we turn to the particular case of a periodically time-dependent interaction which maps to a time-dependent TLS detuning $\varepsilon(t) = \varepsilon_0 + A\cos(\Omega t)$, while higher harmonics do not play a relevant role. In this case the Hamiltonian in Eq. (B9) takes the form

$$H(t) = \frac{\Delta}{2}\sigma_x + \frac{\varepsilon(t)}{2}\sigma_z, \tag{C1}$$

where we have added an irrelevant term proportional to the unit matrix such that $H(t)$ becomes traceless. In the following, we review the typical quantum phenomena observed with this Hamiltonian.

1. Rabi oscillations

The most prominent textbook example is the Rabi problem found for resonant driving with frequency $\Omega \simeq \Delta$, small amplitude $A \ll \Omega, \Delta$ and zero detuning, $\varepsilon_0 = 0$ [7]. In this limit, it is convenient to work in the eigenbasis of the undriven (here uncoupled) system, henceforth denoted by a tilde, which corresponds to the basis of individual pendulum modes. Then the Hamiltonian (C1) reads

$$\widetilde{H}(t) = \frac{\Delta}{2}\sigma_z + \frac{A}{2}\cos(\Omega t)\sigma_x. \tag{C2}$$

In the quantum optical context, this model describes atomic transitions induced by irraditation with a laser that couples to the dipole moment of the atom.

It is now convenient to transform the Hamiltonian via the unitary $S = e^{-i\sigma_z\Omega t/2}$ to a rotating frame, $\widetilde{H} \to S^\dagger \widetilde{H} S - iS^\dagger \dot{S}$. Sufficiently close to resonance and for small driving amplitude, $|\Delta - \Omega| \ll \Omega$ and $A \ll \Omega$, one can neglect within a rotating-wave approximation rapidly oscillating terms to find the time-independent two-level Hamiltonian

$$\widetilde{H}_{\mathrm{eff}} = \frac{1}{2}\begin{pmatrix} \Delta - \Omega & \Omega_\mathrm{R} \\ \Omega_\mathrm{R} & -\Delta + \Omega \end{pmatrix} \tag{C3}$$

with the Rabi frequency

$$\Omega_\mathrm{R} = \frac{A}{2} = \frac{3\mu_0 m_1^2 l_1^2}{\pi\omega_0 J_0 L^5} \simeq \frac{3\mu_0 m_1^2 l_1^2}{\pi M g^2}\,\frac{\omega_0^3}{L^5}\,. \tag{C4}$$

Hence, the occupation probabilities oscillate with the frequency

$$\Omega_{\mathrm{eff}} = \sqrt{(\Delta - \Omega)^2 + \Omega_\mathrm{R}^2}\,. \tag{C5}$$

The latter approximation assumes $J_0 = M\ell^2$ and $\omega_0^2 = g/\ell$ (mathematical pendulum), such that we can eliminate $J_0 \simeq Mg/\omega_0^4$. For resonant driving $\Omega = \Delta$, the resulting dynamics consists of Rabi oscillations between the ground state and the excited state with frequency Ω_R with full probability transfer, i.e., visibility $v = 1$. Thereby, the lower frequency eigenmode of the coupled pendula corresponds to the ground state of the quantum mechanical TLS. Note, that in our Rabi-oscillation experiments no upper magnets are present and, moreover, $A \ll \Delta$, such that also the effective static interaction derived in Sec. A 3 is negligible, $\varepsilon_0 \simeq 0$.

We used Eq. (C5) for the model data shown in Fig. 2c of the main article plotting $\Omega_{\mathrm{eff}}(\Delta)$. In Fig. S2 we additionally compare Eq. (C4) with the measured Rabi frequency while we varied the pivot distance L and the average eigenfrequency of both pendula. For the model curves we used the setup parameters listed in Tables SI and SII. The good agreement between model data and theory in all three cases confirms the validity of our approximations within the regime of small couplings realized in our Rabi measurements.

2. Single Landau-Zener passage

The adiabatic eigenenergies of the Hamiltonian (C1) exhibit an avoided crossing at $\varepsilon = 0$. For large driving amplitudes A, a single traverse of such an avoided crossing is a standard problem in time-dependent quantum dynamics. In

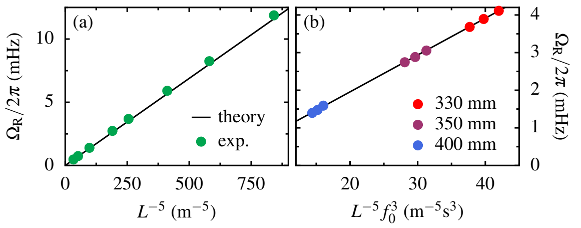

Figure S2. Probing the magnetic dipole-dipole interaction on Rabi oscillations (no upper magnets are used). The symbols are measured Rabi frequencies while the straight lines are calculated with Eq. (C4). The propotionality $\Omega_R \propto L^{-5}$ is a consequence of the interaction energy G_1 in Eq. (A13). Model lines are calculated with material parameters listed in Tables SI and SII, there is no free fit parameter. (a) Rabi frequency as a function of L^{-5} for varying pivot distance L; (b) Rabi frequency as a function of $L^{-5}f_0^3$ for three different L (as labeled) and varying $f_0 = \omega_0/2\pi$, which is the average pendulum eigenfrequency.

an idealized model, one linearizes the time-dependent detuning to obtain $\varepsilon(t) = vt$ with the sweep velocity

$$v = \pm\Omega\sqrt{A^2 - \varepsilon_0^2} \tag{C6}$$

at the crossing. Then the Hamiltonian can be approximated by an idealized version with linearized time-dependence,

$$H_{LZ}(t) = \frac{1}{2}\begin{pmatrix} vt & \Delta \\ \Delta & -vt \end{pmatrix} = \frac{1}{2}\left(vt\sigma_z + \Delta\sigma_x\right). \tag{C7}$$

Since the dynamics takes place close to the crossing, one may extend the time range to infinity, which allows an analytic solution. In 1932, Landau, Zener, Stückelberg, and Majorana [8–11] in four independent works found that if the system at time $t = -\infty$ is in its adiabatic ground state, it will in the limit $t \to \infty$ occupy the excited adiabatic state with the so-called Landau-Zener probabilty

$$P_{LZ} = \exp\left(-\frac{\pi\Delta^2}{2|v|}\right). \tag{C8}$$

In particular for $|v| \ll \Delta^2$, the system adiabatically follows the initialized ground state, while for $|v| \gg \Delta^2$, it non-adiabatically switches to the excited state.

3. LZSM interference

A more recent topic in ac-driven quantum dynamics is the behavior of a quantum system that is repeatedly swept through an avoided crossing. Then each crossing acts as beam splitter in energy space, such that one observes interference patterns as a function of the average detuning ε_0 and the driving amplitude A [12]. Since the crossing condition $\varepsilon(t) = 0$ can only be fulfilled for sufficiently large amplitude, $A \gtrsim |\varepsilon_0|$, a nontrivial interference pattern is found in the triangle that meets this condition during the modulation, cf. Fig. S3. We calculated the interference pattern displayed in Fig. S3(a) based on the linearized Newton's equation, while for the one in Fig. S3(b) we used the Schrödinger equation (B9) which results from a slowly-varying envelope approximation. The LZSM fan diagram computed with Newton's equation contains a clearly visible distortion for large values of ε_0 and A, which is missing in the corresponding calculation using the Schrödinger equation. These distortions are related with the reduced potential curvatures discussed in Sec. B 3 above. Moreover, in the upper-right angle of Fig. S3(a), we observe a region of saturate amplitude pointing to an instability. It emerges when the coupling exceeds the pedulum frequencies, $\varepsilon(t) \gtrsim \omega_0$ at least at some instances of time, such that one eigenvalue of the linearized Newton equation (Eq. (1) of the main text) becomes imaginary. Both, the distortion and the instability pose a limit to a one-to-one comparison between coupled pendula and a quantum mechanical two-level-system.

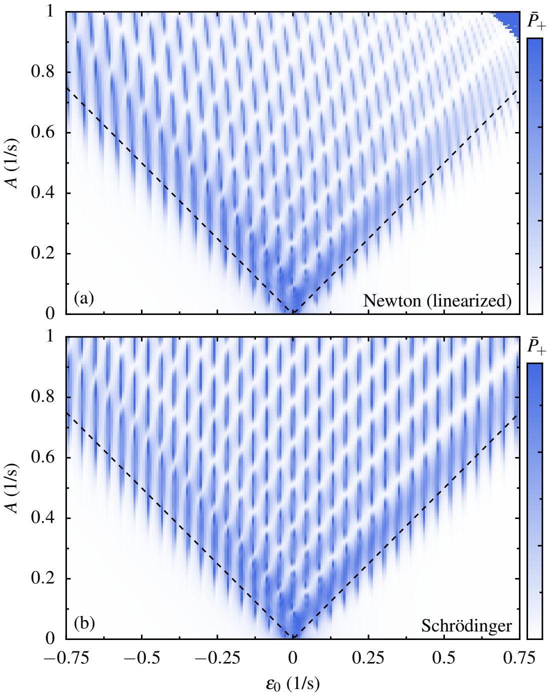

Figure S3. LZSM fan diagram presenting $\overline{P}_+(\varepsilon_0, A)$ computed (a) with the linearized Newton's equation and (b) with the slowly varying envelope approximation which has the form of a Schrödinger equation. The dashed lines indicate $A = |\varepsilon_0|$, below which the avoided crossing at $\varepsilon(t) = 0$ is never reached during the modulation.

We demonstrate a classical analog to a qubit within this limitation [demonstrated by the differences between Fig. S3(a) and Fig. S3(b)]. Beyond, we provide clear evidence for LZSM interference in a macroscopic classical system. In quantum systems, LZSM interference patterns of this kind have been observed for the non-equilibrium population of superconducting qubit [13–18], the current through double quantum dots [6, 19–21], and the response of a cavity coupled to a double quantum dot [22–24].

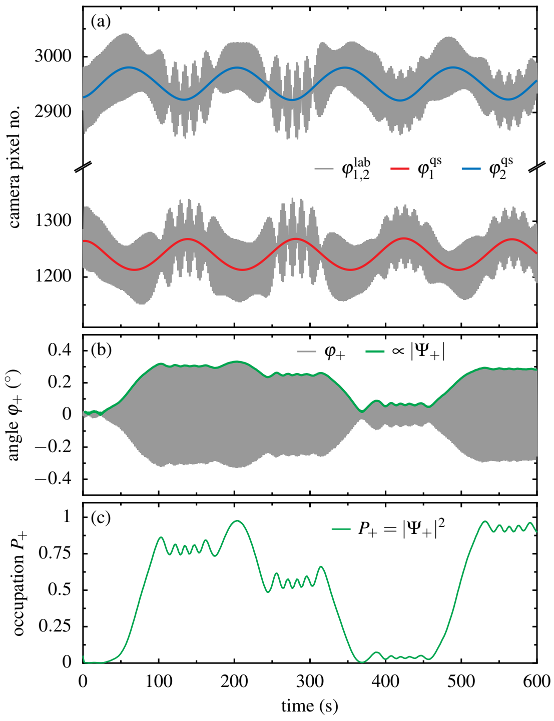

Figure S4. Determining the population dynamics from raw data. (a) Measured horizontal positions (corresponding camera pixels) as a function of time for both pendula during a LZSM experiment (gray). Apart from an offset, they are proportional to the pendulum angles used as labels in the figure. Individual oscillations are not resolved. The quasistatic solutions (colored solid lines) are determined by applying a numerical low-pass filter to the raw data. (b) In-phase mode $\varphi_+(t) = [\varphi_1(t) + \varphi_2(t)]/2$ (gray) as determined from the raw data in panel a after subtracting the $\varphi^{\rm qs}_k(t)$. The green line is the envelope $|\Psi_+|$. (c) Occupation probability $P_+ = |\Psi_+|^2$.

Appendix D: Data analysis

1. Processing of experimental data

The dynamics of the pendula covers a broad spectrum of time scales, dominated by the fastest one, the oscillation of the pendula near their resonances with $\omega_0/2\pi \sim 0.5\,$Hz. The modulation of the interaction with a frequency $\Omega/2\pi \sim 5\,$mHz defines a much slower time scale. A central quantity in our experiments is the modulated interaction quantified by $(G_{\rm u} + G_{\rm l})/2\pi\omega_0 J_0$. It defines the time scale of an envelope of the pendula oscillations, which is slow compared to ω_0. In case of the Rabi experiments it is the smallest time scale, while in our Landau-Zener experiments the modulation is the smallest time scale. In both cases, the resulting beating dynamics can be described by a Schrödinger-like equation and, consequently, can be compared with the dynamics of a qubit.

In Fig. S4(a) we present the horizontal positions of both pendula as a function of time directly measured with the line scan camera visible in the background of Fig. S1. From these, it is straightforward to calculate the deflection angles, $\varphi^{\rm lab}_k$, via a geometric relation. The approximately 300 pendula oscillations with angular frequencies close to ω_0 are not resolved in Fig. S4(a), which spans a time duration of 10 minutes. We decompose these motions into the rapid oscillations φ_k, centered at some unknown value ξ_k, which by and large is the adiabatic equilibrium position $\varphi^{\rm qs}_k$, i.e., we set

$$\varphi^{\rm lab}_k = \varphi_k + \xi_k \tag{D1}$$

with $k = 1,2$. The $\varphi^{\rm qs}_k(t)$ are shown as colored solid lines in Fig. S4(a). Owing to the driving, they oscillate with the slow modulation frequency Ω.

In a first step of processing the data, we determine ξ_k using the fact that its dynamics is much slower than the bare pendula oscillations. On the average, over a few periods of the pendula oscillations, the $\varphi_k(t)$ vanish, such that $\overline{\varphi^{\rm lab}_k} = \xi_k(t)$. Hence, subtraction of this time average from the measured deflection angles $\varphi^{\rm lab}_k(t)$ yields the rapid oscillations

$$\varphi_k(t) = \varphi^{\rm lab}_k(t) - \overline{\varphi^{\rm lab}_k}. \tag{D2}$$

Provided the well separated timescales, a convenient way for performing the time average is a convolution of $\varphi^{\rm lab}_k$ with a Gaussian of width $\sim 10/\omega_0$. Note, that the precise width is practically irrelevant.

For our Rabi experiments we then continue analyzing φ_k while for experiments in the Landau-Zener regime we consider $\varphi_\pm = (\varphi_1 \pm \varphi_2)/2$. In Fig. S4(b) we plot $\varphi_+(t)$ determined from the raw data shown in Fig. S4(a). As the fast oscillations near ω_0 are not resolved in this plot, the data appear as a gray region with modulated height.

The envelope of this modulation, which is a consequence of the driving, is $|\Psi_+|$. To actually determine the envelope dynamics of $\varphi_k(t)$, we square both sides of Eq. (B3) such that the right-hand side becomes $2|\Psi_k|^2$ plus two terms that oscillate with angular frequency $2\omega_0$. These rapidly oscillating terms can be removed by convolution with a Gaussian as described above, which provides

$$2|\Psi_k(t)|^2 = \overline{|\varphi_k|^2}, \tag{D3}$$

where $k = 1,2,+,-$. Notice that with this procedure, we cannot obtain the phases of Ψ_k, hence cannot determine $|\Psi_\pm|$ from $|\Psi_{1,2}|$ or vice versa. Instead, both must be computed from φ_k with Eq. (D3).

To emphasize the correspondence to the probability amplitude of a qubit, in Fig. S4(c) we show the "occupation probability" $P_+(t) = |\Psi_+(t)|^2$ normalized, such that $P_1 + P_2 = P_+ + P_- = 1$. It visualizes the energy transfer between the diabatic modes $\varphi_\pm$ for four subsequent passages through their avoided crossing, while we modulated the magnetic coupling between the pendula. The first pronounced step can be interpreted as a standard Landau-Zener transition, while the three subsequent steps are heavily influenced by the phase development between the pendula oscillations. In between the pronounced steps occur oscillations on a faster time scale, also visible in the individual pendula oscillations in Fig. S4(a). The time scale of these beats corresponds to the (modulated) coupling between the pendula. While the coupling clearly exceeds the modulation frequency in our LZSM experiments, a

look at Fig. 2 of the main paper clarifies, that in the case of Rabi experiments the modulation frequency exceeds the coupling.

2. Effective two-level-system parameters

To obtain numerical data from the Schrödinger equation (B9), we need to know the effective parameters of the driven TLS, namely Δ, ε_0 and A. While the frequency detuning follows readily from the oscillation frequencies of the uncoupled pendula, $\Delta = \omega_1 - \omega_2$, the interaction parameters A and ε_0 require more effort. A straightforward but tedious strategy is based on the Newton equation of the setup with all relevant quantities such as the center of mass, the moments of inertia, the distance between the pivots and the magnetic moments. The effective TLS parameters can then be approximated via a Taylor expansion at the equilibrium position.

An additional difficulty is related with our choice of using magnetic dipoles to generate an interaction between the pendula. On the one hand, it allows us to conveniently modulate the coupling, on the other hand the dipole-dipole interaction gives rise to higher-order terms in the expansion of the interaction potential, discussed above in Sec. A 3. For example, in the absence of the upper magnets, Eq. (B6) predicts for the TLS the driving amplitude $\tilde{A} = G_1/\omega_0 J_0$, where the tilde indicates that we ignore the time-dependence of the potential curvature as in Eq. (A12). Moreover, together with Eq. (A16), it implies that the static TLS detuning ε_0 (i.e. the time-averaged coupling of the pendula) stemming from the third-order term of the potential relates to the TLS driving amplitude according to

$$\varepsilon_0 = \frac{5\tilde{A}^2}{4\omega_0}, \qquad A = \tilde{A}. \tag{D4}$$

While this expression provides the correct order of magnitude, comparison with our experimental data reveals that it overestimates ε_0 substantially (by up to 40%).

To improve our prediction, we refine the above approach by directly evaluating the effective interation G in Eq. (A16) together with the quasi static position (A17) without Taylor expansion of the denominator. For convenience, as in the Hamiltonian (C1), we express the interaction energy in terms of the uncorrected TLS parameter $\tilde{\varepsilon}(t) = \tilde{G}(t)/\omega_0 J_0$, where we approximated the eigenfrequencies and moments of inertia of the pendula by their averages ω_0 and J_0, respectively. Then the quasi static position reads

$$\delta\varphi^{\mathrm{qs}} = \frac{2\tilde{F}(t)/\omega_0 J_0}{\omega_0 - 2\tilde{\varepsilon}(t)}, \tag{D5}$$

while Eq. (A16), which expresses the effective $G(t)$ in terms of the uncorrected $\tilde{G}(t)$, can be replaced by an improved relation between the effective interaction $\varepsilon(t)$ and the uncorrected interaction $\tilde{\varepsilon}(t)$,

$$\varepsilon(t) = \tilde{\varepsilon}(t)\Big(1 - \frac{l_1}{L}\delta\varphi^{\mathrm{qs}}\Big)^{-5}. \tag{D6}$$

If we again approximate the time dependence by its symmetrized form, $\varepsilon(t) = \varepsilon_0 + A\cos(\Omega t)$, we obtain the effective parameters given by the first two Fourier coefficients of $\varepsilon(t)$, namely

$$\varepsilon_0 = \int_0^T \frac{dt}{T}\varepsilon(t), \tag{D7}$$

$$A = 2\int_0^T \frac{dt}{T}\varepsilon(t)\cos(\Omega t). \tag{D8}$$

While the prediction of A and ε_0 from Eq. (D6) surpasses that of Eq. (D4), it still uses an expansion which looses accuracy with increasing coupling strength between the pendula. To circumvent this problem, below we follow an alternative approach, where we determine A and ε_0 from the measured dynamics. This experimental approach is still based on the conjecture that the observed dynamics can be described by the Schrödinger equation (B9) with $\varepsilon(t) = \varepsilon_0 + A\cos(\Omega t)$. In the following, we describe three complementary methods by which this task may be performed and explain, why the third method provides the most accurate results.

a. Fourier analysis

A rather direct method to determine A and ε_0 from measurements is based on the Fourier spectra of the diabatic modes $\varphi_\pm$ or, equivalently, $\Psi_\pm$. It works best, if $\varepsilon(t) > \Delta$ most of the time such as in LZSM experiments. Then the Hamiltonian is dominated by the interaction $\varepsilon(t)$, while the detuning Δ can be neglected. Under such conditions, the mode Ψ_+ remains practically constant, such that its spectrum is dominated by a sharp maximum at zero frequency. In contrast, the time evolution of the out-of-phase mode is given by the phase factor $\Psi_- = e^{iB(t)}$, where $\dot{B} = \varepsilon(t)$. In Fourier space, it becomes the integral

$$\Psi_-(\omega) = \int dt\, e^{i\omega t + iB(t)}, \tag{D9}$$

which we evaluate within the stationary phase approximation. This means that we replace the integral by its contributions in the vicinity of times at which the time derivative of the exponent vanishes, i.e., when the equation $\omega = -\dot{B} = -\varepsilon(t)$ is fulfilled. As ε is bounded, the spectrum is essentially restricted to the frequency range $-\max(\varepsilon) \le \omega \le -\min(\varepsilon)$. The contribution of each stationary point is proportional to $\sqrt{2\pi/\dot{\varepsilon}(t_n)}$, where $\dot{\varepsilon}$ is the second time derivative of the phase in Eq. (D9). This time derivative vanishes at the extrema of $\varepsilon(t)$, such that the $\Psi_-(\omega)$ diverges at the margins of the spectrum. For the assumed shape of the driving, we expect pronounced maxima near $-\varepsilon_0 \pm A$.

There is no need to remove the phase factor $e^{-i\omega_0 t}$ as in the ansatz (B3), because it only shifts the Fourier spectrum to higher frequencies. Thus, in Fig. S5 we performed the analysis directly with the spectra of $\varphi_\pm$ measured for a typical LZSM measurement. Thin lines correspond to the numerically Fourier transformed measured $\varphi_\pm$. While φ_+ yields a peak at the already known value ω_0, the spectrum of φ_- is

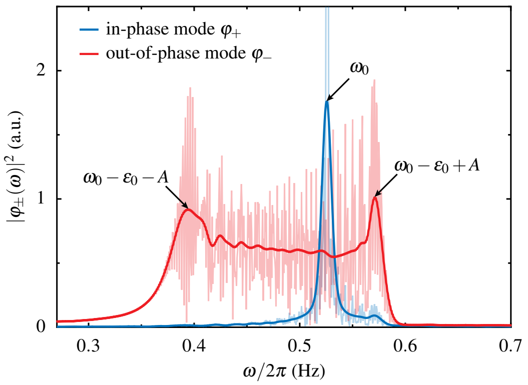

Figure S5. Spectrum of the in-phase and out-of-phase modes for a typical LZSM experiment. The faint lines are the numerical Fourier transforms of the raw data, while the thicker lines are the same data but smoothened with a Gaussian of width $\sigma = 0.025\,\text{Hz}$. The maxima marked by arrows allow us to roughly estimate the detuning ε_0 and the driving amplitude A as discussed in the text.

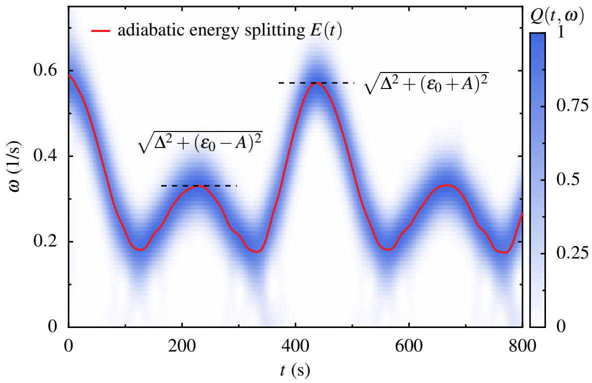

Figure S6. Husimi representation $Q(t)$ (blue scale) of the out-of-phase mode $\Psi_-(t)$ with its spectrum shown in Fig. S5. The average (red solid line) provides an estimate of the time evolution of the splitting $E(t) = \sqrt{\Delta^2 + \varepsilon(t)^2}$. It's maxima allow one to estimate ε_0 and A, see text.

broadened due to the driving and develops a frequency comb spectrum with two main maxima, one near $\omega_0 - \varepsilon_0 - A$ and a second one near $\omega_0 - \varepsilon_0 + A$. To determine these maxima we convolved the experimental spectra with a Gaussian resulting in the fat lines. The positions of the maxima of the out-of-phase mode provide a rough estimate of the parameters ε_0 and A.

Unfortunately, the positions of the maxima of the out-of-phase mode spectrum are not exactly at $\omega_0 - \varepsilon_0 \pm A$. In fact, this Fourier analysis method to determine A and ε_0 systematically underestimates A. In principle, this systematic error could be corrected for in a calibration based on a comparison with alternative methods discussed below.

b. *Husimi analysis*

The next method is capable of determining the full time dependence of $\varepsilon(t)$ by employing a phase-space method frequently used for visualizing semi classical structures of a quantum mechanical wave function $\varphi(x)$. It consists of a mapping of $\varphi(x)$ to a function $Q(x,p)$, whose structure marks the corresponding classical orbits (x_t, p_t). As such it provides the momentum as a function of the position, $p(x)$, with a resolution limited by the uncertainty principle.

Replacing the phase space coordinates (x,p) by time and frequency, one obtains a mapping of a function of time, $\Psi(t)$, to $Q(t,\omega)$. Accordingly, $Q(t,\omega)$ provides the time-resolved oscillation frequency $\omega(t)$. For a two-level system described by the Hamiltonian (B9), the relevant frequency scale is the splitting between the eigenmodes $\omega(t) = E(t) = \sqrt{\Delta^2 + \varepsilon(t)^2}$. Thus, while the time-frequency Husimi representation of $\Psi_+(t)$ is constant at zero, that of $\Psi_-(t)$ traces the adiabatic splitting $E(t)$. In Fig. S6 we plot the Husimi representation of the out-of-phase mode $\Psi_-(t)$ for the data shown in Fig. S5.

The Husimi function can be defined as the modulus squared of the overlap of a function with a wave packet centered at position t and oscillating with a frequency ω,

$$w_{t,\omega}(t') = \exp\Big(-\frac{(t-t')^2}{2\sigma^2} - i\omega t'\Big), \tag{D10}$$

where the width σ shifts the uncertainty towards time (large σ) or frequency (small σ). Thus, $Q(t,\omega) = |q(t,\omega)|^2$, where

$$\begin{aligned} q(t,\omega) &= \int dt'\, w^*_{t,\omega}(t')\Psi(t') \\ &= e^{-i\omega t} \int dt'\, w^*_{0,\omega}(t-t')\Psi(t'). \end{aligned} \tag{D11}$$

The convolution form obtained with the second line is convenient for the numerical evaluation, while the phase factor does not affect $Q(t,\omega)$.

The interpretation by which we motivated the use of the Husimi function becomes evident when one considers a function $\Psi(t) = e^{-iE(t)t}$ with some slowly varying function $E(t)$, such that dE/dt can be neglected. Evaluating the integral in Eq. (D11) within steepest descent, we have to determine the stationary points at which the t'-derivative of the exponent vanishes. Real and imaginary part of this condition read $t' = t$ and $\omega = E(t)$, which means that the structure of $Q(t,\omega)$ is indeed dominated by the momentary oscillation frequency.

The price of the Husimi analysis are the fundamental restrictions of its resolution resulting in a broadening of $E(t)$ in time. As the Fourier analysis above, also this method is based on the evaluation of a Fourier integral within a stationary-phase approximation. As a consequence, it equally suffers from an underestimation of the splitting $E(t)$ and at its turning points and, hence, from an underestimation of A.

The main benefit of the Husimi analysis is its ability to directly visualize the time evolution of the modulation of the splitting $E(t)$ and, hence, the time-dependent coupling strength $|\varepsilon(t)| = \sqrt{E(t)^2 - \Delta^2}$, where the minimal splitting is given by the frequency difference Δ.

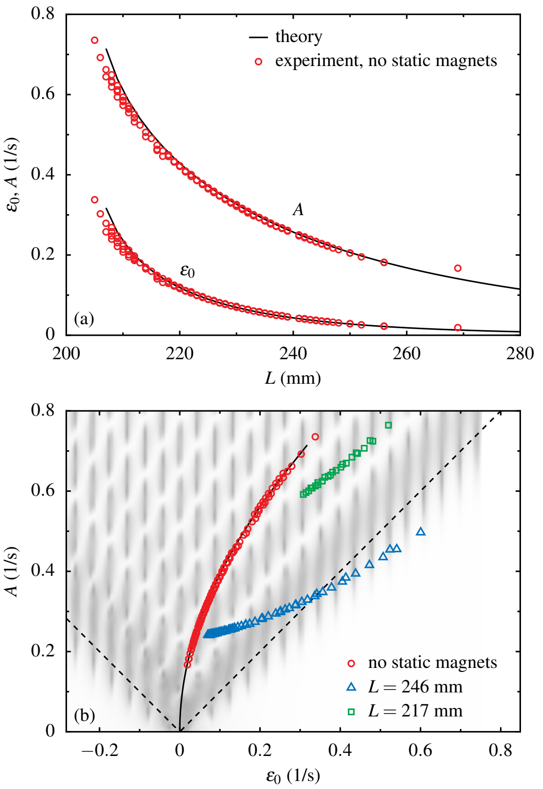

Figure S7. (a) Effective TLS modulation parameters, $A(L)$ and $\varepsilon_0(L)$, reconstructed from the LZSM dynamics without upper magnets for modulation period $T = 441$ s. The red circles result from the analysis of the quasistatic solution $\varphi_{1,2}^{\mathrm{qs}}(t)$ using Eq. (D12), while the solid lines corresponds to the numerical solution based on Eq. (D6). The spectral analyses (Fourier or Husimi analyses, not shown) yield comparable results within their respective accuracies. (b) LZSM interference pattern (gray scale) adopted from Fig. S3b calculated with the Schrödinger equation. The red circles depict $A(\varepsilon_0)$ without upper magnets determined from pairs of data points shown in panel a with varying L, the solid line shows the according prediction. Other symbols show $A(\varepsilon_0)$ for measurements with constant $L = 217$ mm (squares) or $L = 246$ mm (triangles) while we varied the distance L_u between the now present upper magnets.

c. Analysis of the quasistatic solution

To overcome such uncertainties, in our third method we determine ε_0 and A directly from the measured quasistatic solution $\varphi_{1,2}^{\mathrm{qs}}(t)$ discussed in Sec. A 2. It can be directly measured by rotating a magnet without exciting the pendula otherwise, such that $\varphi_{1,2}(t) = \varphi_{1,2}^{\mathrm{qs}}(t)$. Alternatively, as $\Omega \ll \omega_0$, one can extract $\varphi_{1,2}^{\mathrm{qs}}(t)$ with high accuracy from measurements with oscillating pendula by applying a digital lowpass to separate the quasistatic dynamics from $\varphi_{1,2}(t)$, cf. Sec. D 1. Based on our approximation $\varepsilon = \varepsilon_0 + A\cos(\Omega t)$ we determine the extreme values $\varepsilon_{\mathrm{min}}$ and $\varepsilon_{\mathrm{max}}$ and use

$$\begin{aligned} A &= \frac{1}{2}\left(\varepsilon_{\mathrm{max}} - \varepsilon_{\mathrm{min}}\right) \\ \varepsilon_0 &= \frac{1}{2}\left(\varepsilon_{\mathrm{max}} + \varepsilon_{\mathrm{min}}\right) . \end{aligned} \tag{D12}$$

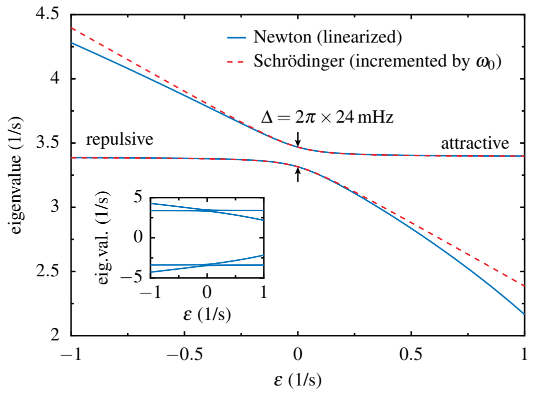

Figure S8. Avoided crossing formed by the eigenfrequencies of the coupled pendula calculated for the detuning of $f_1 - f_2 = 24$ mHz. The solid lines are computed with the linearized Newton's equations, the dashed lines are the eigenvalues of the Schrödinger equation. The inset displays the full spectrum of Newton's equations, including the negative eigenvalues.

The symbols in Fig. S7(a) present the modulation parameters ε_0 and A of the effective TLS as a function of the pivot distance L determined from a series of experiments without upper (static) magnets using Eq. (D12). The solid line corresponds to the numerical solution based on Eq. (D6). The agreement is excellent for $L \gtrsim 220$ mm, while for smaller distances we find noticeable deviations. They indicate, that for our stronger couplings a quantitative derivation of the interaction parameters of the effective TLS from the nonlinear dipole-dipole interaction has its limitations. For this reason, we use the experimental values $\varepsilon_0(L)$ and $A(L)$ for our further analysis.

In Fig. S7(b) we present as a grayscale the LZSM interference pattern already shown in Fig. S3(b), which we calculated numerically based on the Schrödinger equation. On top we plot the values of $A(\varepsilon_0)$ determined from our experiments as symbols. The open circles correspond to the $\varepsilon_0(L)$ and $A(L)$ values shown in Fig. S7(a) for the case of no upper magnets. The solid line behind these points is the prediction based on Eq. (D6) and corresponds to the solid lines in Fig. S7(a). Triangles and squares show experimental values for measurements including upper magnets. In these experiments the distance between the pivots was constant while the distance between the upper magnets was varied. These $A(\varepsilon_0)$ curves are the basis for the comparison of the measured and predicted LZSM interference patterns presented in Figs. 4c and 4d of the main article.

d. Consistency of our approximations

Our approximations are based on the following concept: Small oscillations of a classical many-body system can be described by a linearized equation of motion of the form $M\ddot{\boldsymbol{x}} = V\boldsymbol{x}$, where the coordinate vector $\boldsymbol{x}$ consists of all deviations from the equilibrium position. The symmetric matrix V contains the potential curvatures, while M is the diagonal matrix of the masses of each particle. Since the masses are

positive, $M^{1/2}$ is real valued, such that the equation of motion can be written as $\ddot{y} = -Qy$ with the symmetric matrix $Q = M^{-1/2}VM^{-1/2}$, which has real and non-negative eigenvalues (see Ref. [1] or another text book on classical mechanics). Their square roots are the eigenvalues of the linearized equations of motion and are shown in the inset of Fig. S8.

As a consistency check for our mapping to a Schrödinger equation we verify that its spectrum corresponds to the one of the linearized classical equation of motion. Since we neglect the lower half of the spectrum in the rotating-wave approximation, our results compare to the positive eigenfrequencies of Newton's equation. In addition, our ansatz (B3) corresponds to a gauge transformation that shifts the eigenfrequencies by $-\omega_0$. Hence, the spectrum predicted by our Schrödinger equation shifted by ω_0 finally corresponds to the positive eigenfrequencies of Newton's equation. In the main panel of Fig. S8 we present both spectra in direct comparison in the absence of the driving. For sufficiently small interaction ε our mapping is accurate. We expect, that the mapping works equally well for our driven experiments, as we consider slow driving with $\Omega \ll \omega_0$.

Appendix E: Experimental setup and methods

For visualizing the coherent wave mechanics equivalent to the dynamics of an individual qubit, macroscopic pendula have decisive advantages. First, in contrast to nanoscale devices, our pendula are large and have a slow clock speed, such that their dynamics can be observed with bare eyes. Second, the time evolution of a classical and macroscopic device can be obtained from one single experiment, while quantum- or nanosystems require many repeated measurements at various times. A drawback of our macroscopic pendula is, that a continuous modulation of the eigenfrequencies, corresponding to the energy detuning typically modulated in a qubit, is virtually impossible. It would require moving a weight smoothly up and down, for some measurements along the full length of a pendulum rod while it is oscillating. Therefore, in our experiments we keep the frequency detuning fixed and instead modulate the coupling between the pendula. Modulating the detuning or the coupling are mathematically equivalent options, which can be demonstrated by performing a unitary basis transformation in Sec. B 2. The in-phase and out-of-phase modes of our coupled pendula then correspond to the diabatic (or localized) states of a qubit, while the individual pendula eigenfrequencies correspond to its adiabatic eigenstates, cf. Table I in the main article.

In order to be able to modulate the coupling, we replace the usual spring connecting both pendula by permanent magnets connected to each pendulum. We then modulate the coupling by rotating one of the magnets with a constant angular frequency. The setup is presented in Fig. 1 of the main article and can be experienced in the attached movie. Our magnets are cubicles of pressed neodymium powder coated with nickel bought from Webcraft GmbH (www.supermagnete.de).

1. Details of the setup

Each pendulum consists of a one meter long stainless steel rod with a diameter of 12 mm, extended at the bottom end with a stainless steel thread and a hollow polyethylen housing containing AA batteries, which can drive a linear rotating motor via a simple circuit board. Two cubic neodymium magnets with 28 mm edge length are glued to the axes of each motor. In the experiments discussed here, we rotate one of the two magnets with constant angular frequency. At a distance of 0.513 m above them are two smaller magnets fixed by plastic screws inside plastic cylinders [red in the photograph in Fig. S1(b)] to the rods. These magnets can be moved horizontally inside the cylinders. Brass nuts fixed to the opposite ends of the cylinders function as counter weights to balance the center of masses of the pendula within the respective rods. Heavy brass cylinders (2.14 kg) are screwed onto the threads attached to the pendula. The vertical positions of these weights serve for adjusting the resonance frequencies of the individual pendula. The overall weight of each pendulum is 4.242 kg. In our experiments the air friction can be neglected compared to the friction of the pivots and the damping related to magnetic induction. The frame supporting the pendula is built from hollow aluminum bars, while the pendula are fixed via their pivots to a massive pair of stainless steel beams spanning the top of the frame. The pivots are professional pendulum clock pivots based on leaf springs provided by the company Erwin Sattler GmbH & Co. KG. Due to their plate geometry the leaf springs strongly suppress unwanted pendula motions others than oscillations in the *x*-*y* plane. The pivots quality is essential for providing high enough and stable quality factors. It is important to run the experiment in a tranquil surrounding, because in particular air flows and vibrations can cause uncontrolled phase shifts of the pendula oscillations. Therefore we have placed the frame supporting the pendula on a massive granite plate in a separate and quiet room in the cellar of the building. The coupling between the pendula is provided by up to four magnets. It is modulated whenever one of the lower magnets is rotated. At the high quality factors of several thousands it is necessary to avoid even tiny contributions to the coupling between the pendula mediated by the supporting frame. Initially this was a problem in our setup, which we prevented by stiffening the frame by adding brackets in its corners and by tightly fixing the frame at one side to an approximately 0.5 m thick brick wall of the building.

2. Requirements, line width and strong coupling

For performing qubit simulating experiments, such as Rabi oscillations or LZSM interferometry, the resonance frequencies of the individual pendula have to be much higher than both, the coupling constant and the frequency difference, while the latter two must be highly tunable. At the same time, the quality factor must be high enough to ensure that the coupling strength exceeds the line widths of the eigenmodes by far. Practically, it is desired that for the duration of the experiment damping effects can be neglected, which is the case in

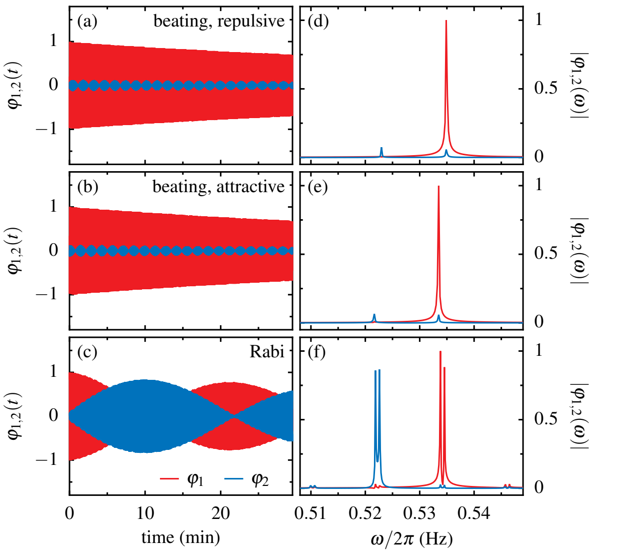

Figure S9. Time evolution of $\varphi_{1,2}$ for beating experiments with maximal repulsive (a) versus maximal attractive (b) couplings and the corresponding resonant Rabi experiment with the coupling modulated at the frequency $\Omega = \Delta$; $L = 454\,$mm, $\Delta/2\pi = 11.7\,$mHz. (d–f), Fourier transforms of the time evolutions shown in panels a–c.

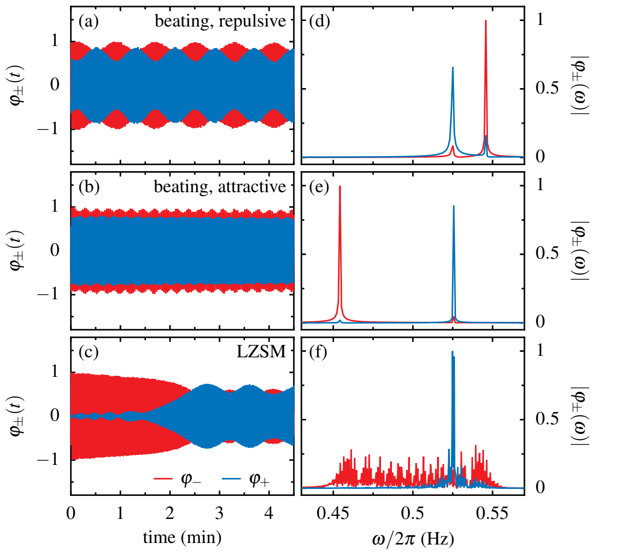

Figure S10. Time evolution of $\varphi_\pm$ for beating experiments with maximal repulsive (a) versus maximal attractive (b) couplings and the corresponding LZSM experiment with the coupling modulated at the frequency $\Omega/2\pi = 2.27\,$mHz; $L = 240\,$mm, $\Delta/2\pi = 6.2\,$mHz. (d–f), Fourier transforms of the time evolutions shown in panels a–c; for the LZSM experiment the Fourier transform in panel f corresponds to five full periods of the modulation, while panel c shows only one period.

our experiments and greatly simplifies the data analysis. The strong distance dependence of the magnetic dipole-dipole interaction provides the desired tunability of the coupling via adjusting the mutual distances between the lower and the upper magnets. Further, rotating one of the magnets allows us to modulate the coupling of the "qubit" analogue. The price is a time dependent momentary equilibrium deflection of the pendulum rods, which is discussed in detail in Appendix A.

If two magnets are moved with respect to each other, their electric conductivity gives rise to eddy currents, which result in the main damping mechanism of our coupled pendula, similar to the functioning of an induction break. The damping is weak, as the Q-factor of our coupled pendula still ranges between $3000-6000$ depending on the average distance of the magnets. With oscillation frequencies $\omega_0/2\pi \sim 0.5\,$Hz, it allows us to observe the qubit equivalent dynamics for several hours. More importantly, our large Q-factors allow us to ignore damping effects within a limited time window $\delta t \ll 2\pi Q/\omega_0$, which facilitates a one-to-one comparison with a quantum mechanical two-level system.

To resolve the splitting of a two-level-system it needs to exceed the line widths $\gamma = \omega_0/2Q$ of the eigenmodes, $\sqrt{\Delta^2+\varepsilon^2} > \gamma$. For our $Q > 3000$ we find $\gamma < 0.6\,\text{ms}^{-1}$ in our experiments. The splitting between the eigenmodes can then be easily resolved by using frequency detunings $|\Delta| > \gamma$. Moreover, for coupled pendula with such a high Q-factor it is straightforward to realize the so-called strong coupling regime defined by $\varepsilon > \gamma$, which is reached in most of our experiments.

For achieving a meaningful comparison between our classical system and a qubit we require a clean separation between the individual pendulum frequencies and all other time scales $\Omega, \Delta, A \ll \omega_0$. We used the modulation frequencies $\Omega/2\pi = 2.3\,$mHz, $\Omega/2\pi = 7.1\,$mHz or $\Omega/2\pi = 11.7\,$mHz, modulation amplitudes of the coupling between $0.7\,\text{mHz} \lesssim A/2\pi \lesssim 43\,$mHz, and frequency detunings $|\Delta|/2\pi \leq 5\,$mHz. To adjust the latter, we re-positioned 2 kg weights along the pendulum rods.

Appendix F: Measurement regimes

1. Rabi experiments

Rabi oscillations can be observed in the limit of small couplings and if the much larger frequency detuning is similar to the modulation frequency, $\Delta \sim \Omega \gg \Omega_R = A/2$, cf. Sec. C 1. For simplicity we performed our Rabi experiments without upper magnets, such that (for small couplings) $A \simeq \varepsilon_{\max}$. Practically, our modulation frequencies of a few mHz dictate a range of useful frequency differences Δ and couplings $A < \Delta$, the latter being controlled by the distance L between the pivots.

In Figs. S9(a) and S9(b) we present the deflections of both pendula $\varphi_k(t)$ for beating experiments without driving ($A = 0$, $\varepsilon = \varepsilon_{\min}$ or $\varepsilon = \varepsilon_{\max}$) for the two extreme coupling cases with the magnets aligned either antiparallel for maximal repulsion or collinear for maximal attraction, where the pivot distance $L = 454\,$mm corresponds to a small coupling. To initialize each measurement, we deflected just one of the two pendula, the one corresponding to the red lines. The energy transfer between the two pendula is clearly incomplete owing to the finite frequency detuning, $\Delta/2\pi = 11.7\,$mHz, while the beating frequency is $\sqrt{\Delta^2+\varepsilon^2}/2\pi \simeq \Delta/2\pi$. The latter corresponds to the

difference between the respective eigenfrequencies, directly visible in the Fourier spectra shown in Figs. S9(d) and S9(e). The spectrum of the pendulum that was initially not deflected (blue) clearly contains two maxima, where the frequency of the smaller peak coincides with the main maximum of the initially deflected pendulum (red). This indicates a finite mixing between the states represented by φ_1 and φ_2, which are not the exact eigenmodes because of the coupling between the pendula. Note, that the eigenfrequencies are slightly smaller for the attractive interaction as compared to the case of repulsive interaction.

In Fig. S9(c) we present the corresponding resonant Rabi experiment with identical parameters as above but the coupling being modulated with the angular frequency $\Omega = \Delta$. In this case, the initial beating experiments mark the turning points of the modulation of the coupling during the Rabi experiment. The energy transfer between the two pendula is now complete but happens at the Rabi frequency $\Omega_R/2\pi \simeq 0.7\,\text{mHz}$, hence the initially postulated condition $\Omega_R \ll \Delta, \Omega$ is fulfilled.

The Fourier spectra of the Rabi experiment in Fig. S9(f) reveal two main peaks and four side peaks for each pendulum. The main peaks are splitted by the (effective) Rabi frequency in Eq. (C5). The much smaller side peaks, each of which is equally split by the (effective) Rabi frequency, are higher order components split off by multiples of Ω from the main peaks. In the resonant case $\Omega = \Delta$, the frequency values of the Fourier components of both pendula coincide, in the non-resonant case they would be displaced by $\Omega - \Delta$. Note that the higher order components in the Fourier spectra are responsible for the weak stepwise modulation with frequency Ω of the occupation of the pendula, which are weakly visible in Fig. S9(c).

2. LZSM experiments

For a direct comparison with the small coupling regime of Rabi experiments we perform similar measurements within the regime of LZSM experiments at much larger modulation of the coupling with $A > \Omega$. We consider measurements for $L = 240\,\text{mm}$, $\Delta/2\pi = 6.2\,\text{mHz}$, $\Omega/2\pi = 2.27\,\text{mHz}$ and a sizable ε_0 realized by including upper magnets. For such large couplings ($A, |\varepsilon_0| \gg \Delta$) the diabatic modes $\varphi_\pm$ approximately correspond to the eigenmodes. Hence, in Fig. S10 we now plot $\varphi_\pm$. The beating experiments, which we again performed for collinear versus antiparallel magnets, summarized in the upper four panels of Fig. S10 reveal the expected much larger range of couplings compared to the Rabi experiment. The very different beating frequencies for attractive versus repulsive interactions point to a sizable ε_0. Note, that the frequency (main component of Fourier spectrum) of φ_+ is almost identical for repulsive versus attractive coupling (blue in Figs. S10d and S10e), while the frequency of φ_- (red) varies by roughly 20 %.

The LZSM experiment, presented by its first avoided crossing in Fig. S10(c), shows the expected energy transfer between the in-phase and out-of-phase modes near the avoided

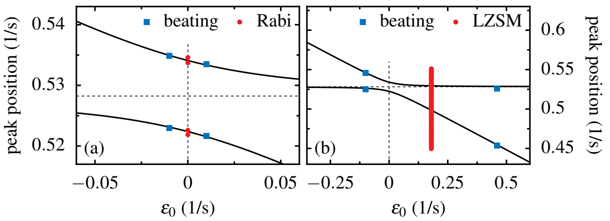

Figure S11. Avoided crossings (solid lines) with Fourier components of beating experiments (blue squares, $\Omega = 0$) and main Fourier components of modulated experiments (red circles, $\varepsilon = \varepsilon_0$). (a) Rabi experiment for $f_1 = 0.53335\,\text{Hz}$, $f_2 = 0.52165\,\text{Hz}$, $L = 454\,\text{mm}$ and $\Omega/2\pi = 11.7\,\text{mHz}$. (b) LZSM experiment for $f_1 = 0.5290\,\text{Hz}$, $f_2 = 0.5228\,\text{Hz}$, $L = 240\,\text{mm}$ and $\Omega/2\pi = 2.27\,\text{mHz}$.

crossing. The additional faster beats vary in frequency related with the time dependence of ε. The Fourier spectrum plotted in Fig. S10(f) comprises five modulation periods. It reveals that the in-phase mode stays at the frequency of the beating experiments, while the out-of-phase mode contains frequency components spanning a slightly larger region than that between the out-of-phase mode frequencies of the beating experiments. Note, that the apparent splitting, say $\delta\omega$, of the in-phase mode φ_+ in the Fourier spectrum of the LZSM experiment resembles a slight difference between the frequencies that the in-phase mode has between attractive versus repulsive beating experiments. As a result, the the out-of phase mode spectrum is composed of two copies of a frequency comb with the same relative shift $\delta\omega$, each one characterized by equally spaced peaks separate by the modulation frequency Ω.

3. Avoided crossings for Rabi versus LZSM experiments

Figure S11 summarizes the main components of the Fourier spectra of the experiments presented in Figs. S9 and S10 and thereby visualizes the vastly different experimental regimes realized in a Rabi experiment, where $A < \Delta$, versus a LZSM experiment with $A \gg \Delta$.

To highlight the differences between the regimes of the Rabi versus LZSM experiments, in Figs. S11(a) and S11(b) we plot for the Rabi versus LZSM experiments shown in Figs. S9 and S10 the relevant regions of the avoided crossings predicted for the measured frequencies by the Schrödinger equation (for the time independent quantum mechanical two-level system). The blue squares indicate the components of the respective Fourier spectra of the beating experiments, where we determined the values of ε using the eigenvalue equation $\omega_\pm = \frac{1}{2}(-\varepsilon \pm \sqrt{\Delta^2 + \varepsilon^2})$. The red circles in Fig. S11(a) indicate the four main components of the Fourier spectra of the Rabi experiment in Fig. S9(f), where we used $\varepsilon = \varepsilon_0 \simeq 0$ for simplicity. The line of red circles in Fig. S11(b) indicates the range of the frequency comb of the Fourier spectrum of φ_- of the LZSM experiment, cf. Fig. S10(f), where we again used $\varepsilon = \varepsilon_0$ for simplicity.

Clearly, for the presented Rabi experiment in Fig. S11(a), $\varepsilon_0 \simeq 0$ and $A \ll \Delta$, while for the LZSM experiment in Fig.

S11(b), both $\varepsilon_0, A > \Delta$. This depicts the main difference between the two regimes.

Appendix G: Notations, units and magnitudes

Tables SI–SIII below summarize the variables as well as their magnitudes used in the main text and in the supplement.

Table SI. Variables of individual pendula

variable, definition	explanation	values
$\varphi_{1,2}$	deflection angles of individual pendula	$\lvert\varphi_{1,2}\rvert < 0.8^\circ \simeq 0.014\,\mathrm{rad}$
M	overall mass of each pendulum	4.242 kg
g	acceleration due to gravity in Munich (PTB table)	9.807232 m/s^2
$f_1 \mid \omega_1 = 2\pi f_1$	resonance | angular frequency of pendulum 1	$f_1 \simeq (0.52-0.55)\,\mathrm{Hz}$
$f_2 \mid \omega_2 = 2\pi f_2$	resonance | angular frequency of pendulum 2	$f_2 = 0.52195\,\mathrm{Hz}$
$l_{\mathrm{r1}} = g/\omega_1^2$	reduced length of pendulum 1	(0.818 - 0.912) m
$l_{\mathrm{r2}} = g/\omega_2^2$	reduced length of pendulum 2	0.912 m
l_{c1}	center of mass of pendulum 1 (distance from pivot)	$(0.754-0.841)\,\mathrm{m}$
l_{c2}	center of mass of pendulum 2 (distance from pivot)	0.841 m
$J_1 = M l_{\mathrm{c1}} l_{\mathrm{r1}}$	moment of inertia of pendulum 1 (respective pivot)	(2.619 - 3.254) kg m^2
$J_2 = M l_{\mathrm{c2}} l_{\mathrm{r2}}$	moment of inertia of pendulum 2 (respective pivot)	3.254 kg m^2
$Q_{1,2}$	quality factor of individual uncoupled pendula	$9500-10500$
The following distances are equal for both pendula:		
l_{p}	distance between pivot and point of measurement	1.053 m
l_{l}	distance between pivot and center of lower magnet	1.148 m
l_{u}	vertical distance between pivot and center of upper magnet	0.635 m

Table SII. Variables related with the coupling between the pendula

variable, definition	explanation	values and units
$\varphi_\pm = (\varphi_1 \pm \varphi_2)/2$	in-phase — out-of-phase mode	$< 0.8^\circ$
L	distance between pivots, i.e., lower magnets for $\varphi_1 = \varphi_2 = 0$	$(0.205-0.454)\,\mathrm{m}$
m_{l}	magnetic moment of each lower magnet	25.37 Am^2
L_{u}	distance between upper magnets for $\varphi_1 = \varphi_2 = 0$	$(0.105-0.168)\,\mathrm{m}$
m_{u}	magnetic moment of each upper magnet	6.544 Am^2
$J_0 = (J_1 + J_2)/2$	mean moment of inertia	$\simeq 3\,\mathrm{kg\,m^2}$
$\omega_0 = (\omega_1 + \omega_2)/2$	mean angular frequency of both pendula	$(3.28-3.37)\,\mathrm{s^{-1}}$
$\Delta = \omega_1 - \omega_2$	angular frequency difference	$(0-29)\times 10^{-3}\mathrm{s^{-1}}$
$\varepsilon = G/\omega_0 J_0$	coupling constant	$(-0.4-1.3)\,\mathrm{s^{-1}}$
$G(G_{\mathrm{l}}, G_{\mathrm{u}})$	effective potential curvature (see Sec. A 3)	
$G_{\mathrm{l}} = 6\mu_0 m_{\mathrm{l}}^2 l_{\mathrm{l}}^2/\pi L^5$	interaction energy between lower magnets	$(0.1-5.6)\,\mathrm{J}$
$G_{\mathrm{u}} = 6\mu_0 m_{\mathrm{u}}^2 l_{\mathrm{u}}^2/\pi L_{\mathrm{u}}^5$	interaction energy between upper magnets	$(0.31-3.2)\,\mathrm{J}$
Q	quality factor of coupled pendula (for realized range of L)	$3000-6000$

Table SIII. Additional variables related with the modulation of the coupling

variable, definition	explanation	values and units
$2\pi/\Omega$	period of magnets' rotation	85.5 s, 141 s, 441 s
$\varepsilon(t) = \varepsilon_0 + A\cos\Omega t$	modulated coupling constant (linear approximation)	$[(-0.4)-(+1.3)]\,\mathrm{s^{-1}}$
$\Omega_{\mathrm{R}} = G_{\mathrm{l}}/2\omega_0 J_0 = A/2$	rabi frequency for $\Omega_{\mathrm{R}} \ll \omega_0, \Delta$ and if only lower magnets are used	$(4.5-265)\times 10^{-3}\mathrm{s^{-1}}$
$P_{\mathrm{LZ}} = \exp(-\pi\Delta^2/2\lvert v\rvert)$	Single passage Landau-Zener probability	$0-1$
$v = \frac{d\varepsilon(t)}{dt}\rvert_{\varepsilon=0} = \pm\Omega\sqrt{A^2-\varepsilon_0^2}$	Speed of driving at avoided crossing at $\varepsilon = 0$	$(\pm 0.0027 - \pm 0.0300)\,\mathrm{s^{-2}}$
P_0	Initial probability to occupy in-phase mode	$0-0.2$

[1] H. Goldstein, C. Poole Jr., and J. Safko, *Classical Mechanics*, 3rd ed. (Pearson, San Francisco, 2001).

[2] J. D. Jackson, *Classical Electrodynamics*, 3rd ed. (Wiley, New York, 1999).

[3] M. J. Seitner, H. Ribeiro, J. Kölbl, T. Faust, J. P. Kotthaus, and E. M. Weig, Classical Stückelberg interferometry of a nanomechanical two-mode system at room temperature, Phys. Rev. B **94**, 245406 (2016).

[4] B. W. Shore, M. V. Gromovyy, L. P. Yatsenko, and V. I. Romanenko, Simple mechanical analogs of rapid adiabatic passage in atomic physics, Am. J. Phys. **77**, 1183 (2009).

[5] L. Novotny, Strong coupling, energy splitting, and level crossings: A classical perspective, Am. J. Phys. **78**, 1199–1202 (2010).

[6] F. Forster, G. Petersen, S. Manus, P. Hänggi, D. Schuh, W. Wegscheider, S. Kohler, and S. Ludwig, Characterization of qubit dephasing by Landau-Zener-Stückelberg-Majorana interferometry, Phys. Rev. Lett. **112**, 116803 (2014).

[7] C. Cohen-Tannoudji, J. Dupont-Roc, and G. Grynberg, *Atom photon interaction: Basic processes and applications* (Wiley, New York, 1992).

[8] L. D. Landau, Zur Theorie der Energieübertragung bei Stößen, Phys. Z. Sowjetunion **2**, 46 (1932).

[9] C. Zener, Non-adiabatic crossing of energy levels, Proc. R. Soc. London A **137**, 696 (1932).

[10] E. C. G. Stueckelberg, Theorie der unelastischen Stösse zwischen Atomen, Helv. Phys. Acta **5**, 369 (1932).

[11] E. Majorana, Atomi orientati in campo magnetico variable, Nuovo Cimento **9**, 43 (1932).

[12] S. N. Shevchenko, S. Ashhab, and F. Nori, Landau-Zener-Stückelberg interferometry, Phys. Rep. **492**, 1 (2010).

[13] W. D. Oliver, Y. Yu, J. C. Lee, K. K. Berggren, L. S. Levitov, and T. P. Orlando, Mach-zehnder interferometry in a strongly driven superconducting qubit, Science **310**, 1653 (2005).

[14] M. Sillanpää, T. Lehtinen, A. Paila, Y. Makhlin, and P. Hakonen, Continuous-time monitoring of Landau-Zener interference in a Cooper-pair box, Phys. Rev. Lett. **96**, 187002 (2006).

[15] C. M. Wilson, T. Duty, F. Persson, M. Sandberg, G. Johansson, and P. Delsing, Coherence times of dressed states of a superconducting qubit under extreme driving, Phys. Rev. Lett. **98**, 257003 (2007).

[16] A. Izmalkov, S. H. W. van der Ploeg, S. N. Shevchenko, M. Grajcar, E. Il'ichev, U. Hübner, A. N. Omelyanchouk, and H.-G. Meyer, Consistency of ground state and spectroscopic measurements on flux qubits, Phys. Rev. Lett. **101**, 017003 (2008).

[17] D. M. Berns, M. S. Rudner, S. O. Valenzuela, K. K. Berggren, W. D. Oliver, L. S. Levitov, and T. P. Orlando, Amplitude spectroscopy of a solid-state artificial atom, Nature (London) **455**, 51 (2008).

[18] J. Li, M. P. Silveri, K. S. Kumar, J.-M. Pirkkalainen, A. Vepsäläinen, W. C. Chien, J. Tuorila, M. A. Sillanpää, P. J. Hakonen, E. V. Thuneberg, and G. S. Paraoanu, Motional averaging in a superconducting qubit, Nature Comm. **4**, 1420 (2013).

[19] J. Stehlik, Y. Dovzhenko, J. R. Petta, J. R. Johansson, F. Nori, H. Lu, and A. C. Gossard, Landau-Zener-Stückelberg interferometry of a single electron charge qubit, Phys. Rev. B **86**, 121303(R) (2012).

[20] E. Dupont-Ferrier, B. Roche, B. Voisin, X. Jehl, R. Wacquez, M. Vinet, M. Sanquer, and S. De Franceschi, Coherent coupling of two dopants in a silicon nanowire probed by Landau-Zener-Stückelberg interferometry, Phys. Rev. Lett. **110**, 136802 (2013).

[21] F. Forster, M. Mühlbacher, R. Blattmann, D. Schuh, W. Wegscheider, S. Ludwig, and S. Kohler, Landau-Zener interference at bichromatic driving, Phys. Rev. B **92**, 245422 (2015).

[22] J. V. Koski, A. J. Landig, A. Pályi, P. Scarlino, C. Reichl, W. Wegscheider, G. Burkard, A. Wallraff, K. Ensslin, and T. Ihn, Floquet spectroscopy of a strongly driven quantum dot charge qubit with a microwave resonator, Phys. Rev. Lett. **121**, 043603 (2018).

[23] X. Mi, S. Kohler, and J. R. Petta, Landau-Zener interferometry of valley-orbit states in Si/SiGe double quantum dots, Phys. Rev. B **98**, 161404(R) (2018).

[24] M.-B. Chen, B.-C. Wang, S. Kohler, Y. Kang, T. Lin, S.-S. Gu, H.-O. Li, G.-C. Guo, X. Hu, H.-W. Jiang, G. Cao, and G.-P. Guo, Floquet state depletion in ac-driven circuit QED, Phys. Rev. B **103**, 205428 (2021).
